# Orphan nuclear receptor 4A1 (NR4A1) and NR4A2 are endogenous regulators of CD71 and their ligands induce ferroptosis in breast cancer

**DOI:** 10.1038/s41419-025-08143-5

**Published:** 2025-11-03

**Authors:** Arafat Rahman Oany, Srijana Upadhyay, Wai Ning Tiffany Tsui, Amanuel Hailemariam, Sarah Latka, John D. Landua, Sandra D. Scherer, Alana L. Welm, Hugo Villanueva, Michael T. Lewis, Stephen Safe

**Affiliations:** 1 Department of Veterinary Physiology and Pharmacology, College of Veterinary Medicine, Texas A&M University, College Station, TX USA; 2 Advanced Technology Cores, Baylor College of Medicine, Houston, TX USA; 3 Breast Center, Baylor College of Medicine, Houston, TX USA; 4 Department of Oncological Sciences, Huntsman Cancer Institute, University of Utah, Salt Lake City, UT USA; 5 Otolaryngology – Head and Neck Surgery, Baylor College of Medicine, Houston, TX USA

**Keywords:** Breast cancer, Molecular biology

## Abstract

Ferroptosis is an iron-dependent cell death pathway that involves multiple genes, including the transferrin receptor (TFRC/CD71), glutathione peroxidase 4 (GPX4) and cystine-glutamate antiporter (SLC7A11). This study is based on the hypothesis that orphan nuclear receptor 4A1 (NR4A1) and NR4A2 maintain low levels of ferroptosis in triple negative breast cancer (TNBC) cells and bis-indole derived (CDIM) compounds act as NR4A1/2 ligands that induce ferroptosis by enhancing CD71 expression. 1,1-Bis(3′-indolyl)-1-(3,5-disubstitutedphenyl)methane (DIM-3,5) analogs were investigated for their cytotoxicity and effects on NR4A1 and NR4A2 regulated genes and induction of ferroptosis. Several assays also determined enhanced lipoperoxidation, reactive oxygen species and malondialdehyde formation in TNBC cells. Knockdown of NR4A1, NR4A2, Sp1 and Sp4 was carried out by RNA interference. Molecular mechanisms of NR4A1/2-mediated regulation of CD71 expression were determined using CD71-luciferase promoter constructs, overexpression of Sp1 and chromatin immunoprecipitation (ChIP) assays. Initial studies show that DIM-3,5 analogs act as an inverse NR4A1/NR4A2 agonists that downregulate the pro-oncogenic responses/gene products regulated by both receptors in TNBC cells. DIM-3,5 analogs also induced ROS, malondialdehyde and lipoperoxide formation in TNBC cells, and this was accompanied by decreased expression of GPX4 and SLC7A11 and induction of CD71. Induction of CD71, an important biomarker of ferroptosis was observed after treatment of TNBC cells with DIM-3,5 analogs, knockdown of NR4A1, NR4A2, Sp1 or Sp4 demonstrating that induction of CD71 was coregulated by both receptors. Moreover, both promoter and ChIP analysis indicated that NR4A1 and NR4A2 acted as ligand-dependent cofactors of Sp1/4-mediated expression of CD71 in TNBC cells. Thus, CD71, a key biomarker of ferroptosis is an NR4A1/2/Sp regulated gene that can be directly targeted by DIM-3,5 inverse NR4A1/2 agonists to induce ferroptosis in TNBC cells.

## Introduction

The orphan nuclear receptor 4A1 (NR4A) sub-family members NR4A1 (Nur77), NR4A2 (Nurr1) and NR4A3 (Nor1) are stress/inflammation-induced immediate-early genes that play key functional roles in maintaining cellular homeostasis and in pathophysiology [1, 2]. In inflammation and stress-induced diseases, such as solid tumors NR4A1 is overexpressed and is a negative prognostic factor for lung, ovarian, colon and breast cancer patients [3–6]. NR4A2 is also highly expressed in solid tumors and is a negative prognostic factor for cervical, gastrointestinal, prostate, nasopharyngeal, colorectal, breast, gastric and pancreatic cancer patients [7–14]. Moreover, NR4A1 and NR4A2 knockdown result in decreased growth, migration and increased apoptosis in most solid tumors, whereas the functional role and prognostic significance of NR4A3 is variable [15]. Studies in this laboratory have identified bis-indole derived compounds (CDIMs) that bind both NR4A1 and NR4A2, and these include 1,1-bis(3′-indolyl)-1-(4-hydroxyphenyl)methane (DIM-4-OH) and 1,1-bis(3′-indolyl)-1-(4-chlorophenyl)methane (DIM-4-CI), respectively [16, 17]. Structure-activity studies have identified a series of 3,5-disubstitutedphenyl CDIM (DIM-3,5) analogs that inhibit mammary tumor growth in athymic nude mice bearing MDA-MB-231 cells at doses <1 mg/kg/day [18] and the highly potent DIM-3,5 compounds have recently been identified as unique dual NR4A1/2 ligands that bind both NR4A1 and NR4A2, and act as inverse agonists in cancer cells [19]. Moreover, DIM-3,5 ligands also decrease PD-L1 expression [20] and enhance CD8+/CD4 + T cell ratios in tumor infiltrating lymphocytes in a syngeneic orthotopic mouse model of breast cancer [18, 19].

Breast cancer is one of the most commonly diagnosed tumors in women and improved therapies coupled with increased awareness of this disease has resulted in a decrease of annual deaths from breast cancer [21]. However, treatment modalities for triple negative breast cancer (TNBC), a highly aggressive form of this disease have only had limited success [22]. Tumors from patients with TNBC do not express the estrogen receptor (ERα, ESR1), progesterone receptor (PR) or epidermal growth factor receptor 2 (ERBB2^+^), and the overall lack of prime molecular targets has negatively impacted the identification of drugs for successfully treating TNBC. In recent years, there has been increasing interest in the development of ferroptosis-inducing agents for treating TNBC; this type of lipoperoxide-induced cell death is dependent on multiple oxidative stress-induced pathways and obviates the need for targeting more well-established drug targets [23–25]. Several natural products with anticancer activities induce ferroptosis in TNBC and other cell lines, and these include quercetin, tetrandrine, flavonoids and other polyphenolics [26–29]. Recent studies show that quercetin, tetrandrine and flavonoids bind NR4A1 and inhibit NR4A1-dependent pro-oncogenic pathways and genes in breast and other cancer cell lines [30–33]. Based on our previous in vivo studies with DIM-3,5 dual NR4A1/2 ligands [18, 19], we hypothesize that NR4A1/2 may be important drug targets in TNBC due, in part, to their regulation of ferroptosis and key ferroptotic genes. This manuscript addresses the hypothesis and demonstrates for the first time that the transferrin receptor (CD71) is an NR4A1/2-regulated gene that can be directly targeted by DIM-3,5 ligands to induce ferroptosis in TNBC.

## Results

### Role of NR4A1 and NR4A2 as pro-oncogenic factors in TNBC

**Fig. 1:**
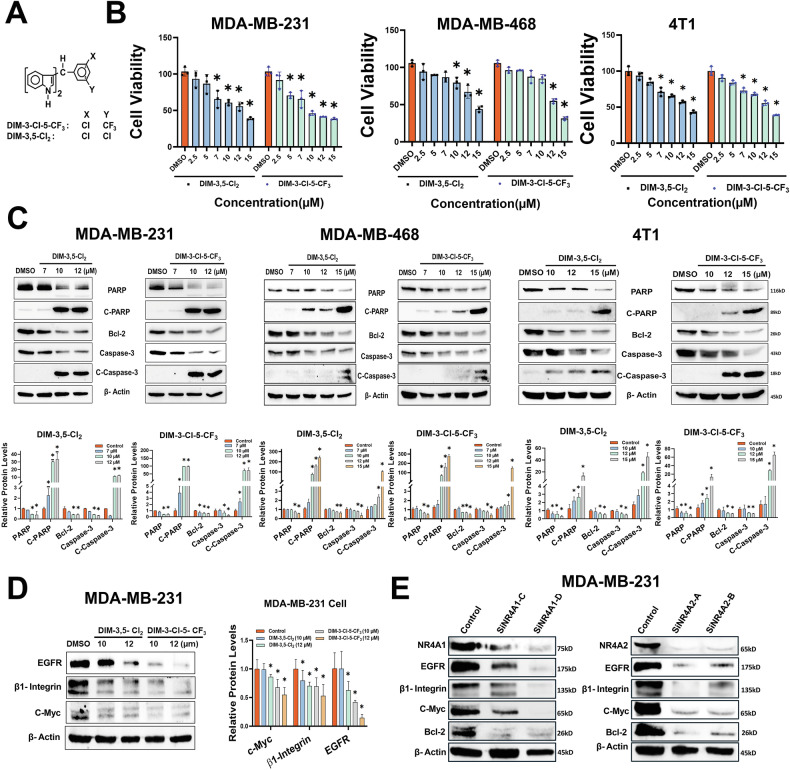
Effects of DIM-3,5 ligands and receptor knockdown on TNBC growth and selected gene products. **A** Structures of DIM-3,5 compounds. **B** MDA-MB-231, MDA-MB-468 and 4T1 cell were treated with DMSO and DIM-3,5 compounds and effects on cell viability were determined as outlined in the “Methods”. **C** MDA-MB-231, MDA-MB-468 and 4T1 cells were treated with DIM-3,5 compounds (ranging from 7 to 15 µM/L) for 24 h, and whole cell lysates were analyzed by Western blots and quantified, as outlined in the “Methods”. MDA-MB-231 cells were treated with DMSO and DIM-3,5 compounds (10 and 12 µM/L) (**D**) for 24 h or transfected with oligonucleotides targeting NR4A1 (siNR4A1) or NR4A2 (siNR4A2) (**E**) and whole cell lysates were analyzed by Western blots as outlined in “Methods”. Cell viability studies were carried out in triplicate, and results are expressed as means ± SD, and significant (*p* < 0.05) inhibition, and induction/reduction are indicated (*).

Initial studies used DIM-3,5 analogs containing 3,5-dichlorophenyl (DIM-3,5-CI_2_) and 3-chloro-5-trifluoromethylphenyl (DIM-3-CI-5-CF_3_) groups and show that both compounds inhibited viability of human MDA-MB-231, MDA-MB-468 and mouse 4T1 TNBC cells (Fig. 1B). Treatment with DIM-3,5 compounds also induced cleaved PARP and cleaved caspase-3, and also decreased BCL-2, PARP, and caspase-3 protein expression in MDA-MB-231, MDA-MB-468 and 4T1 (Fig. 1C) demonstrating the proapoptotic activity of DIM-3,5 compounds that bind both receptors and act as inverse agonists [15, 18]. Previous studies have characterized several NR4A1-regulated genes, including *EGFR*, *β*1*-integrin*, *c-Myc* and *bcl-*2 that are downregulated after NR4A1 knockdown or treatment with CDIM compounds [15]. Using MDA-MB-231 cells as a model, both DIM-3,5-CI_2_ and DIM-3-CI-5-CF_3_ decreased expression of these gene products (Fig. 1C (MDA-MB-231)-D), and similar results were observed after knockdown of NR4A1 or NR4A2 by RNA interference (Fig. 1E). Thus, like NR4A1, NR4A2 also regulates expression of a similar set of pro-oncogenic gene products, and this is consistent with the downregulation of these proteins by DIM-3,5 dual NR4A1/2 inverse agonists.

### Dual NR4A1/2 ligands induce ferroptosis in TNBC cells

**Fig. 2:**
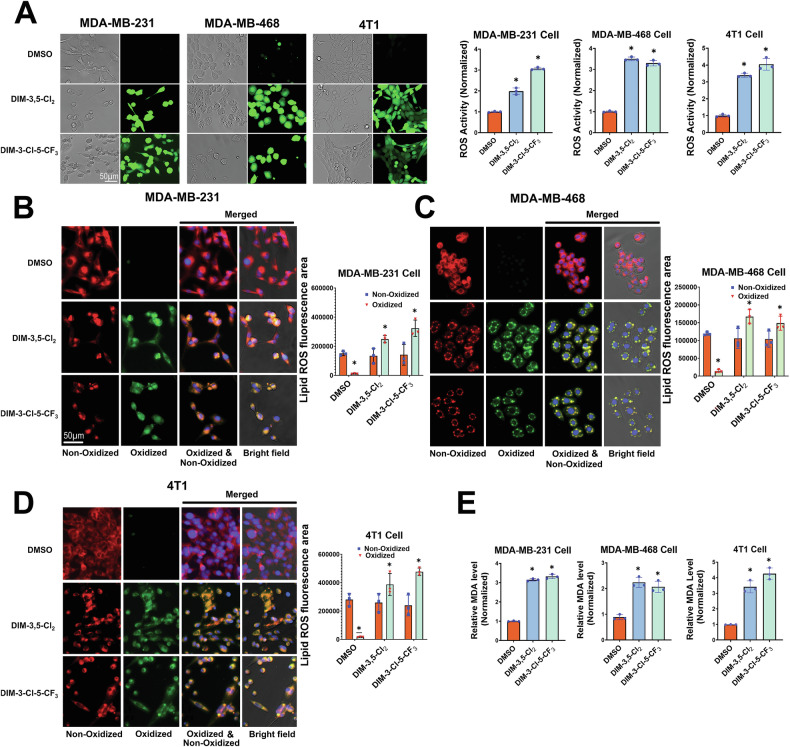
Induction of ROS induced lipoperoxidation by DIM-3,5 compounds. TNBC cells were treated with DMSO and DIM-3,5 compounds at a concentration of 12 µM/L for 16 h, and ROS (**A**), lipoperoxidation (MDA-MB-231 (**B**), MDA-MB-468 (**C**), and 4T1 (**D**) cells) and MDA (**E**) were determined and quantitated fluorometrically as outlined in the “Methods”. Results **A**–**E** are means ± SD of 3 replicate treatments and significant (*p* < 0.05) induction of ROS, MDA formation, and lipoperoxides formation (oxidized control vs treatment) are indicated (*).

Ferroptosis is dependent on the induction of ROS, which is required for Fe^3+^-dependent production of lipoperoxides that result in fatty acid degradation and cell damage. Results in Fig. 2A show that DIM-3,5-CI_2_ and DIM-3-CI-5-CF_3_ significantly induce ROS in MDA-MB-231, MDA-MB-468 and 4T1 cells (16-h treatment) as determined using the cell-permeable H_2_CDCFDA dye. The formation of oxidized lipids is a hallmark of ferroptosis [34, 35] and results summarized in Fig. 2B–D show that DIM-3,5-CI_2_ and DIM-3-CI-5-CF_3_ induce lipoperoxidation in MDA-MB-231, MDA-MB-468 and 4T1 cells using BODIPY™ 581/591 C11 as a sensor of lipoperoxide formation. Quantitation of the results showed that DIM-3,5 ligands enhanced the formation of lipoperoxides (oxidized) (Fig. 2B–D).

Subsequently, lipoperoxidation breakdown induces significant cell damage and fatty acid degradation resulting in the formation of malondialdehyde (MDA) and DIM-3,5-CI_2_ and DIM-3-CI-5-CF_3_ significantly induced MDA formation in the three TNBC cell lines (Fig. 2E)

These results clearly demonstrate that the dual NR4A1/2 ligands induce ferroptosis as evidenced by induction of these diagnostic oxidative pathways, and therefore, we further investigated the role of NR4A1/NR4A2 and the DIM-3,5 analogs on modulation of key ferroptotic genes.

**Fig. 3:**
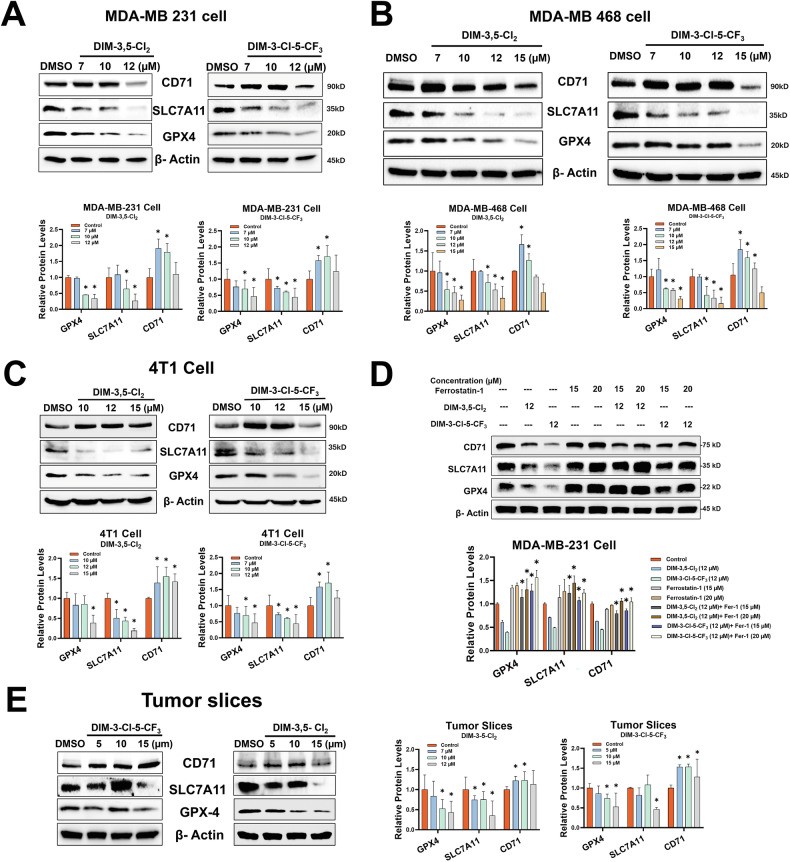
DIM-3,5 compounds modulate expression of ferroptotic gene products in TNBC cells. MDA-MB-231 (**A**), MDA-MB-468 (**B**) and 4T1 (**C**) cells were treated with DMSO or DIM-3,5 compounds (ranging from 7 to 15 µM/L) for 24 h, and whole cell lysates were analyzed by Western blots and quantified to determine changes in key ferroptotic genes. **D** Effect of ferrostatin-1 on DIM-3,5 compounds alone and in combination were determined in MDA-MB-231 cells after treatment for 24 h, and whole cell lysates were analyzed by Western blots and quantified, as outlined in the “Methods”. **E** Tumor slices were obtained as outlined in the “Methods” and after treatment with DIM-3,5 analogs for 72 h whole cell lysates were obtained and analyzed by western blots and quantified, as outlined in the “Methods”. Results are means ± SD for replicate (3) determinations and significant (*p* < 0.05) induction is indicated (*).

Treatment of TNBC cells with DIM-3,5-CI_2_ and DIM-3-CI-5-CF_3_ for 24 h decreased SLC7A11 and GPX4 proteins in MDA-MB-231, MDA-MB-468 and 4T1 cells (Fig. 3A–C, respectively). The concentration-dependent effects of DIM-3,5 compounds on CD71 expression were somewhat variable in the three breast cancer cell lines. In 4T1 cells, CD71 protein levels were slightly decreased after treatment with 15 µmol/L DIM-3,5-Cl_2_ compounds for 24 h, whereas in MDA-MB-231 and MDA-MB-468 cells, 7 and 10 µmol/L DIM-3,5 analogs induced levels of CD71 protein at one or both concentrations but 12 or 15 µmol/L DIM-3,5 downregulated CD71 protein expression. Figure 3D summarizes the effects of the ferroptosis inhibitor ferrostatin-1 on CD71, SLC7A11 and GPX4 expression alone or in combination with 12 µmol/L DIM-3,5 compounds. DIM-3,5 compounds alone decreased expression of CD71, SLC7A11 and GPX4 proteins at the high 12 µmol/L concentration, whereas 15 and 20 µmol/L ferrostatin-1 alone slightly induced SLC7A11 and GPX4 proteins compared to the control and did not affect levels of CD71 protein in MDA-MB-231 cells. In combination studies, ferrostatin-1 significantly inhibited the DIM-3,5-mediated downregulation of all 3 proteins, thus inhibiting the pro-ferroptotic effects of DIM-3,5 analogs on downregulation of GPX4, SLC7A11 and CD71 compared to the treatment alone (Fig. 3D). Similar results were also observed in tumor slices treated with the DIM-3,5 analogs (Fig. 3E).

### DIM-3,5 dual NR4A1/2 ligands induce CD71 expression in TNBC cells

**Fig. 4:**
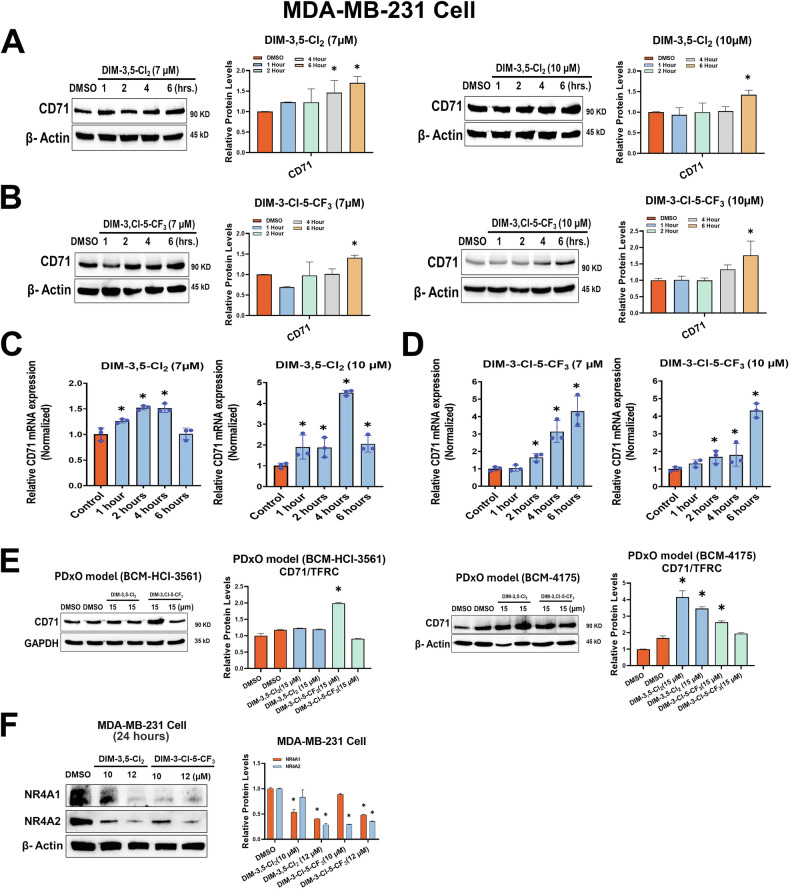
Induction of CD71 by DIM-3,5 compounds. MDA-MB-231 cells were treated with DMSO, 7 and 10 µM DIM-3,5-CI_2_ (**A**) and 7 and 10 µM DIM-3-CI-5-CF_3_ (**B**) for up to 6 h and whole cell lysates were analyzed by Western blots and quantified as outlined in the “Methods”. The time-dependent (0–6 h) induction of CD71 mRNA by 7 and 10 µM DIM-3,5-CI_2_ (**C**) and 7 and 10 µM DIM-3-CI-5-CF_3_ (**D**) was determined by RT-PCR as outlined in the “Methods”. **E** PDxO organoids were treated with 15 µM DIM-3,5 compounds for 24 h, and whole cell lysates were analyzed for CD71 expression by Western blots and quantified as outlined in the “Methods”. **F** MDA-MB-231 cells were treated with DMSO and 10–12 µM DIM-3,5 compounds for 24 h, and whole cell lysates were analyzed by Western blots and quantified as outlined in the” Methods”. Results are means ± SD for replicate (3) determinations and significant (*p* < 0.05) induction is indicated (*).

Overexpression of CD71 is a confirmed biomarker of ferroptosis [36] and the results illustrated in Fig. 4 showed that in MDA-MB-231 cells treatment with 7 and 10 µmol/L DIM-3,5 ligands for 6 h significantly induced CD71 protein levels (Fig. 4A, B) and these results were consistent with the effects of treatment with ≤10 µmol/L DIM-3,5 ligands for 24 h. In which DIM-3,5 ligands are acting as receptor agonists (Fig. 3A).

A similar approach was used for determining the time-dependent induction of CD71 mRNA levels in MDA-MB-231 (Fig. 4C, D). Both 7 and 10 µmol/L DIM-3,5-CI_2_ and DIM-3-CI-5-CF_3_ induced CD71 mRNA levels however, the time courses of the maximal induction responses were variable and compound-dependent. To investigate the effects of DIM-3,5-CI_2_ and DIM-3-CI-5-CF_3_ in pre-clinical 3D models of TNBC, we tested these compounds using NR4A1/2-expressing PDX-derived organoid (PDxO) models and observed induction of CD71 after treatment with 15 µmol/L concentrations of these dual receptor ligands (Fig. 4E). Results illustrated in Fig. 4F show that after treatment of MDA-MB-231 cells with or higher dose (10 or 12 µM) of DIM-3,5 ligands, there was a decrease in both NR4A1 and NR4A2 levels. This correlates with decrease induction of CD71 at higher dose levels and longer treatment times (24 h), which may be due, in part to ligand-induced downregulation of both NR4A1 and NR4A2 after treatment. These results suggest that CD71 expression is induced by the dual NR4A1/2 ligands acting as agonists but downregulated at higher ligand concentration due to receptor downregulation.

### DIM-3,5 analogs activate NR4A1/NR4A2:Sp to induce CD71 in TNBC cells

**Fig. 5:**
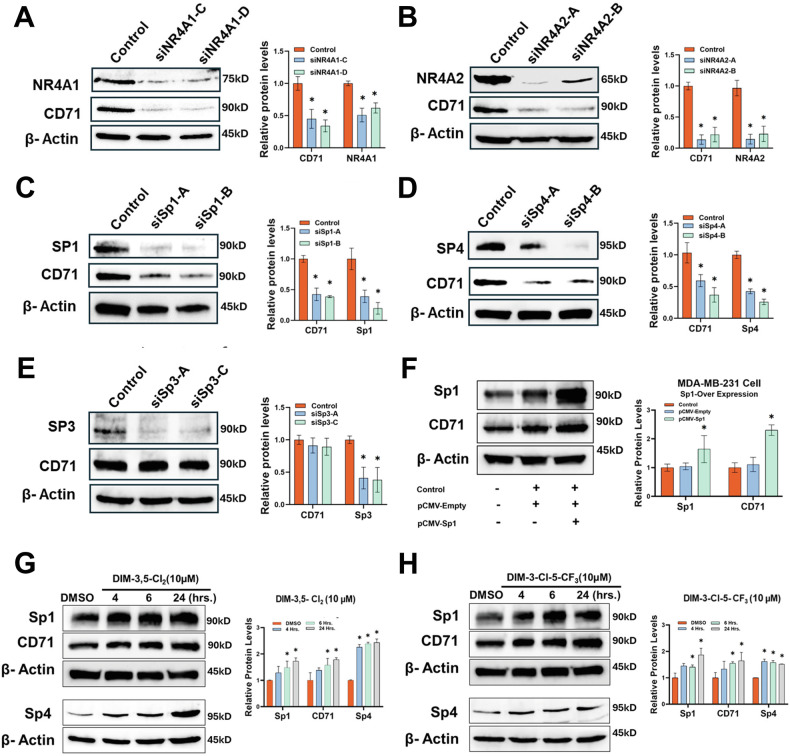
Role of NR4A1 and Sp TFs in the regulation of CD71. MDA-MB-231 cells were transfected with oligonucleotides targeting NR4A1 (**A**), NR4A2 (**B**), Sp1 (**C**), Sp4 (**D**) and Sp3 (**E**), and after 72 h, whole cell lysates were analyzed by Western blots and quantified as outlined in the “Methods”. **F** Effects of Sp1 overexpression on CD71 were obtained by transfection of the p^CVM-Sp1^ expression plasmid and subsequent western blot analysis of whole cell lysates and quantified as outlined in the “Methods”. Cells were treated with 10 µM DIM-3,5-CI_2_ (**G**) and DIM-3-CI-5-CF_3_ (**H**) for up to 24 h, and effects on CD71, Sp1 and Sp4 (separate blot) were determined by Western blots of whole cell lysates and quantified as outlined in the “Methods”.

Results illustrated in Fig. 5A, B show that knockdown of NR4A1 and NR4A2 by RNA interference decreased expression of CD71 in MDA-MB-231 cells demonstrating that both receptors coregulate expression of this key ferroptotic gene. It has previously been reported that Sp1 plays an important role in regulating expression of CD71 through interactions with GC-rich sites in the CD71 gene promoter [37]. Previous studies also show that NR4A1 and other nuclear receptors can act as nuclear cofactors that interact with DNA bound Sp1 to regulate basal gene expression in cancer cells and receptor ligands, such as DIM-3,5 compounds modulate (inhibit or induce) expression of the Sp-regulated genes through a cofactor-dependent pathway [38]. Figure 5C, D illustrate that knockdown of Sp1 and Sp4, but not Sp3 (Fig. 5E), decrease expression of CD71 in MDA-MB-231 cells, suggesting that both NR4A1 and NR4A2 cooperatively act as nuclear cofactors to activate Sp1- and Sp4-dependent CD71 expression. This complements results of previous studies showing that NR4A1 coactivated Sp1 or Sp4, but not Sp3, and in these studies the CDIM-derived NR4A ligands act as inverse agonists to inhibit NR4A1/Sp1:Sp4-mediated expression of PD-L1, PAX3-FOX01, G9a, β1- and several other integrins in cancer cell lines [20, 39–45]. In contrast, the DIM-3,5 dual NR4A1/2 ligands act as receptor agonists for induction of CD71. In addition, the overexpression of the Sp1 (p^CMV-Sp1^) plasmid enhances CD71 protein expression, and this supports the involvement of both NR4A1/2 and Sp1 in regulating CD71 compared to the empty vector (p^CMV-Empty^) as a negative control (Fig. 5F).

Results shown in Fig. 5G, H confirm that treatment with 10 µmol/L DIM-3,5 compounds for 24 h in MDA-MB-231 cells leads to an increase in Sp1 and Sp4 expression, and this is consistent with the enhanced expression of CD71. Results obtained after NR4A1 and NR4A2 knockdown were specific for each receptor and did not affect the expression of NR4A3 (Supplementary Fig. 1).

**Fig. 6:**
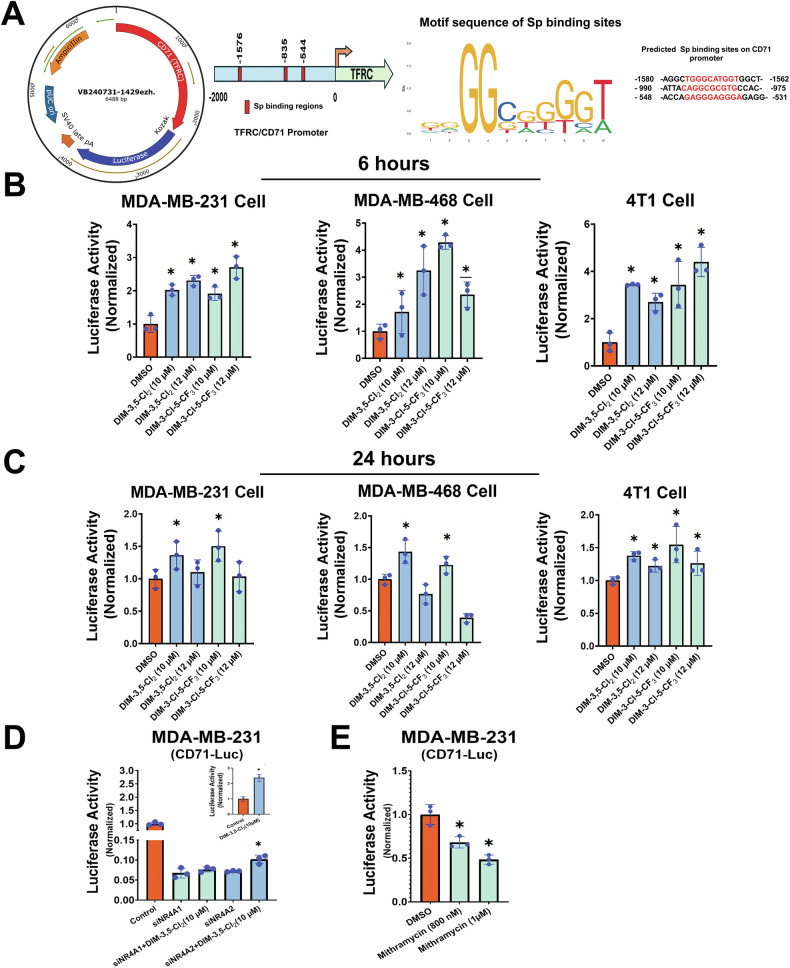
Induction of CD71-luc activity by DIM-3,5 compounds. **A** The vector construct is outlined and −2000 to +200 region of the CD71 promoter was cloned into a mammalian promoter-testing vector (pRP [Pro]-{CD71} > Luciferase) and with predicted Sp binding sites. **B** MDA-MB-231, MDA-MB-468, and 4T1 cells were transfected with CD71-luc construct and treated with DMSO and DIM-3,5 ligands (10–12 µM) for 6 h and luciferase activity was determined as outlined in the “Methods”. **C** MDA-MB-231, MDA-MB-468, and 4T1 cells were transfected with CD71-luc construct and treated with DMSO and DIM-3,5 compounds (10 and 12 µM) for 24 h and luciferase activity was analyzed as outlined in the “Methods”. **D** MDA-MB-231 cells were transfected with or without (insert image) oligonucleotides targeting NR4A1 and NR4A2, and after 48 h cells were transfected with CD71-luc construct and later treated with DMSO and 10 µM DIM-3,5-CI_2_. **E** MDA-MB-231 cells were transfected with CD71-luc construct and treated with DMSO and (800 nM and 1 µM) Mithramycin. Results are expressed as means ± SD for replicate (3) determinations for each treatment group, and significant (*p* < 0.05) induction (compared to DMSO controls) is indicated (*).

We further investigated the induction of CD71 by DIM-3,5 compounds using a CD71-luc construct containing the predicted Sp binding sites (−1576, −835, and −544, respectively of the CD71 promoter (Fig. 6A), and the −1576 to −1566 sequence has previously been identified as important cis elements for regulating CD71 expression [37]. TNBC cells transfected with the CD71-luc construct [pRP [Pro]-{CD71} > Luciferase], and we observed that treatment with DIM-3,5 compounds for 6 h significantly induced luciferase activity (Fig. 6B) cells and this is consistent with previous studies showing that transactivation of CD71 was primarily associated with this region of the promoter [37]. In TNBC cells transfected with the CD71-luc construct treatment with DIM-3,5 analogs for 24 h also induced luciferase activity (Fig. 6C) but in some cells the high concentrations of DIM-3,5 ligands decreased luciferase activity. These results complemented the effects of DIM-3,5 analogs on CD71 mRNA and protein levels in TNBC cells. The roles of NR4A1 and NR4A2 in mediating activity of the CD71-luc construct was further investigated in MDA-MB-231 cells, in which loss of the receptors, decreased luciferase activity and treatment with DIM-3,5-Cl_2_ (10 µmol/L) resulted in minimal induction compared to wild type cells (Fig. 6D). Moreover, Mithramycin, an inhibitor of Sp regulated transcription, also inhibited luciferase activity (Fig. 6E).

**Fig. 7:**
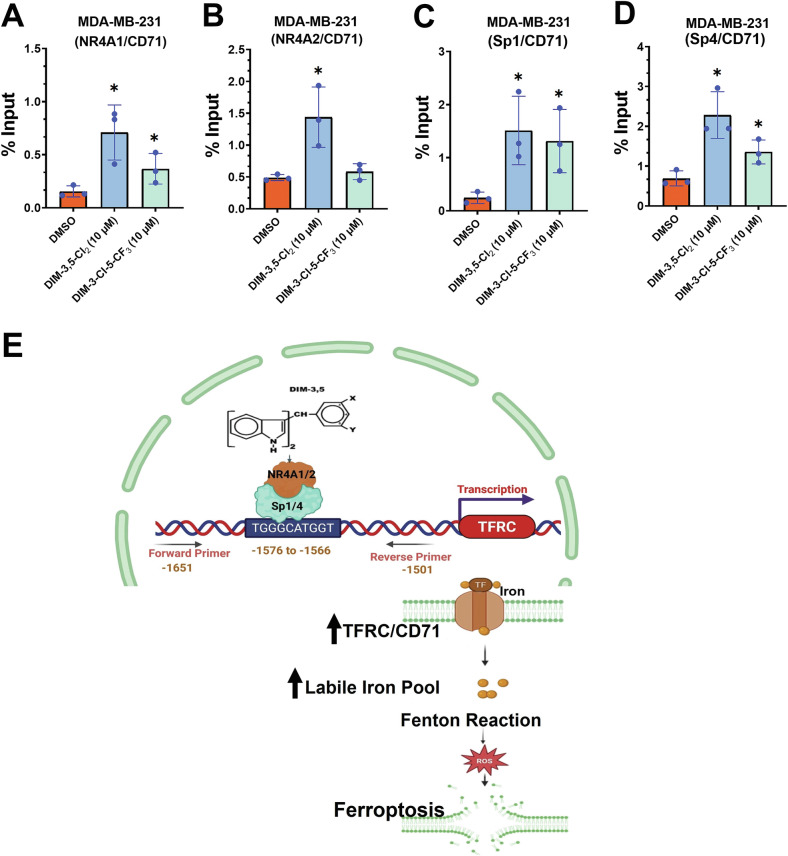
Chromatin immunoprecipitation results in MDA-MB-231 cells. Interactions of NR4A1 (**A**), NR4A2 (**B**), Sp1 (**C**) and Sp4 (**D**) with the CD71 gene promoter containing the Sp binding region was determined after treatment with DIM-3,5 compounds in a ChIP assay (in triplicate) as outlined in the “Methods section”. **E** Summary of the CD71 gene promoter, Sp binding sites (−1576 to −1566) and the primers used for detecting protein interactions in this region of the promoter, along with the overall mechanism of ferroptosis induction. Results are expressed as means ± SD for replicate (3) determinations for each treatment group (**A**–**D**) and significant (*p* < 0.05) induction is indicated (*).

The association of the nuclear factors NR4A1, NR4A2, Sp1 and Sp4 that regulate expression of CD71 with the CD71 gene promoter was further investigated in a ChIP assay using primers targeting three specific binding regions, as shown in Fig. 6A and listed in Supplementary Table 2. Among them, only site one, that encompass the “TGGGCATGGT” active region of the CD71 gene promoter [41] yielded a positive result. RT-PCR analysis of solvent (DMSO)-treated MDA-MB-231 cells demonstrated that NR4A1, NR4A2, Sp1, and Sp4 were associated with this promoter region (Fig. 7A–D). Treatment of MDA-MB-231 with either DIM-3,5-CI_2_ or DIM-3-CI-5-CF_3_ for 6 h primarily increased association of NR4A1, NR4A2, Sp1 and Sp4 with the CD71 promoter. The enhanced ligand-dependent interactions of NR4A and Sp with CD71 promoter correlated with the induction of CD71 by DIM-3,5 analogs. In contrast, for genes, such as PD-L1 that are repressed by DIM-3,5 compounds in TNBC cells [20] there is a decrease of one or more of NR4A1 and Sp bound to the GC-rich PD-L1 promoter, and this is observed in other genes downregulated by NR4A/Sp [38–43]. A proposed model for the induction of CD71 expression by NR4A1 and NR4A2 is depicted in Fig. 7E, and this pathway allows for the direct targeting of this key pro-ferroptotic gene.

## Discussion

Studies in this laboratory have shown that C-DIM compounds act as functional NR4A1 inverse agonists in cancer cells and inhibit TNBC cell and tumor growth and the EC_50_ values for inhibiting tumor growth in athymic nude mice bearing MDA-MB-231 cells by DIM-3,5 analogs is <1 mg/kg/day [18]. This dose was much lower than observed for 4-hydroxyphenyl moiety [DIM-4-OH], an initial prototypic NR4A1 ligand. The potent antitumorigenic activity of the DIM-3,5 analogs are due to several factors, including their activity as dual NR4A1/NR4A2 inverse agonists that inhibit the pro-oncogenic pathway/genes, such as PD-L1, regulated by both receptors in cancer cells [18, 19]. DIM-3,5 analogs also enhance immune surveillance and reverse CD8^+^ and CD4^+^ T cell exhaustion in a syngeneic mouse model of colon cancer [43]. We hypothesized that DIM-3,5 NR4A1/2 ligands also target other receptor-dependent pathways in TNBC and that one of these may be the induction of ferroptosis, an ROS-dependent cell death pathway. This hypothesis was based on previous reports showing that several natural products, including polyphenolics, such as quercetin, tetrandrine and resveratrol all induce ferroptosis in cancer cells [24–29] and it has recently been reported that these compounds also bind NR4A1 and exhibit inverse NR4A1 agonist activity [30–33] similar to that observed for DIM-3,5 analogs [18]. In addition, the NR4A1 ligand DIM-4-OH downregulates expression of stearoyl-CoA-desaturase in pancreatic cancer cells to activate ferroptosis [46]. However, in this study expression of desaturase was minimal in TNBC cells.

DIM-3,5-CI_2_ and DIM-3-CI-5-CF_3_ were recently characterized as ligands that bind both NR4A1 and NR4A2 and inhibit NR4A1/NR4A2-dependent genes and pathways in cancer cells [18, 19, 47] and this was confirmed in TNBC cells as illustrated in Fig. 1. Both compounds inhibited breast cancer cell growth and induced apoptosis and there was a concordance between the effects of NR4A1 and NR4A2 knockdown and effects of DIM-3,5 analogs in TNBC cells and on several NR4A-regulated genes including *EGFR*, *β1-integrin*, *bcl-2* and *c-Myc*. Quercetin, which is an NR4A1 ligand, induces many of these same responses in Rh30 rhabdomyosarcoma cells [32] and also induces ferroptosis in breast and other cancer cell lines [26, 28]. The parallel functions between DIM-3,5 analogs and quercetin can also be extended to their effects on ferroptosis since DIM-3,5-CI_2_ and DIM-3-CI-5-CF_3_ induce ROS, lipoperoxidation and MDA formation and also decrease expression of GPX4 and SLC7A11 in TNBC cell lines and similar results have been observed for other flavonoids in cancer cells [25–29]. CD71 also plays a key role in ferroptosis by facilitating the transport of Fe^3+^ into the cell and the combination of Fe^3+^ ROS can lead to lipoperoxidation [47, 48].

CD71 expression is an important biomarker of ferroptosis [40] and has a primary function of facilitating Fe^3+^ uptake into the cell. DIM-3,5 compounds induce CD71 gene and gene product formation in MDA-MB-231 and other TNBC cells. However, this response is both concentration and time-dependent and high concentrations tend to decrease levels of CD71. Sp1 and Sp4 knockdown resulted in decreased CD71 levels, and this is consistent with previous reports showing that Sp1 interactions with the −1576 to −1566 region of the CD71 gene promoter are important for CD71 expression [37]. However, NR4A1 and NR4A2 knockdown also decreased levels of CD71 protein demonstrating the NR4A1 and NR4A2 cooperatively regulated basal CD71 expression by acting as cofactors of Sp1 and Sp4 transcription factors bound to GC-rich gene promoter elements. Thus, NR4A1 and NR4A2 act as ligand-dependent cofactors of Sp1/4 and this is consistent with results of ChIP assays showing that Sp1/4 and NR4A1/NR4A2 interact with the transcriptionally active GC-rich region of the CD71 promoter, and their expression is either unchanged or induced after treatment with DIM-3,5 compounds. Nuclear receptors, such as NR4A1 and NR4A2 interact with multiple proteins, including DNA bound transcription factors, such as Sp and AP-1 to regulate endogenous expression of multiple genes [38]. For example, ERα/Sp regulates expression of cyclin D1 through interaction with GC-rich sites and ERα agonists and antagonist induce or repress cyclin D1 expression, respectively [38, 49]. Knockdown of NR4A1, NR4A2 or Sp1/4 also decrease expression of NR4A1/Sp-regulated genes, such as PD-L1, β1- and other integrins in breast cancer cells [20, 40, 42] and similar results were observed for CD71 in this study demonstrating that NR4A1 and NR4A2 in combination with Sp1 and Sp4 regulate basal expression of this key ferroptotic gene. Previous studies show that several NR4A1/Sp1-regulated genes are downregulated after treatment with DIM-3,5 ligands, which act as inverse agonists and these effects are accompanied by decreased association of NR4A1, NR4A2 or Sp1/4 with the target gene promoter as determined in ChIP assays [20, 42]. In contrast, the induction of CD71 by DIM-3,5 compounds increased association of one or more of NR4A1, NR4A2, Sp1 or Sp4 with the transcriptionally active GC-rich region of the CD71 promoter. In summary, these results further define the mechanism of CD71 expression and induction in TNBC cells and demonstrate that NR4A1, NR4A2, Sp1, and Sp4 regulate endogenous expression of CD71 via NR4A/Sp complexes, and the overall mechanism may involve the receptors acting as monomers or heterodimers. Future studies will investigate ferroptosis-induced damage associated molecular patterns by DIM-3,5 analogs and their direct effects on immune cells and also their interactions with NR4A1/2 ligand-mediated immune responses.

## Materials and methods

### Cell culture, reagents, ligands and cell proliferation

The breast cancer cell lines MDA-MB-231(CRM-HTB-26) and MDA-MB-468 (HTB-132) from humans and mouse 4T1 (CRL-2539) cancer cells were purchased from American Type Culture Collection (Manassas) and routinely checked for mycoplasma contamination. The maintenance and growth of these cell lines and effects of compound on cell proliferation was carried out as previously described [18, 20, 42].

### Tissue slice and PDxO experiments

The Baylor College of Medicine (BCM) Institutional Animal Care and Use Committee approved all procedures using live animals. Female immune-compromised mice (SCID/beige, Inotiv stock 186) were used to grow the previously established patient-derived xenograft (PDX) model, BCM-15120. Protocols and surgical procedures used to generate BCM-15120 have been described [50, 51]. Tumor disks from PDX were immediately rinsed in sterile PBS and transferred to a 24-well gas permeable tissue culture plate (Coy Laboratory) containing media [52] treated with DIM-3,5-CI_2_ and DIM-3-CI-5-CF_3_; media and compounds were replaced every 24 h, and after 72 h, whole cell lysates of the tumor disks were obtained and analyzed by Western blots. Tumor organoids derived from patient-derived xenografts (PDX) were established as described [52, 53], treated with DIM-3,5-Cl_2_ and DIM-3-Cl-5-CF_3_ for 24 h, and whole cell lysates were analyzed by Western blots [18–20].

### Measurement of ROS

The cell-permeable dye-CM-H_2_DCFDA (Invitrogen, # C6827) was used as an indicator for the detection of reactive oxygen species (ROS) in cells. Human (MDA-MB-231 and MDA-MB-468) and mouse (4T1) breast cancer cells were cultured at a density of 3.0 × 10^5^ cells per well for 24 h, treated with 12 µmol/L DIM-3,5-Cl_2_, DIM-3-Cl-5-CF_3_ and DMSO for 16 h, and ROS was determined according to the manufacturer’s protocol and as described [54].

### Lipid peroxidation and malondialdehyde (MDA) assays

The BODIPY™ 581/591 C11 (Life Technologies, # D3861) was used for determination of lipoperoxidation. The fluorescence properties of this probe shifted from red signals (581/610 nm) to green signals (484/510 nm) upon oxidation of the polyunsaturated butadienyl segment of fatty acids in live cells. Breast cancer cells were treated with DMSO 12 µmol/L, DIM-3,5-Cl_2_, and DIM-3-Cl-5-CF_3_ for 16 h and using the BIODIPY™ probe cells were analyzed for lipoperoxidation according to the manufacturer’s protocol. MDA was measured and quantitated using the Lipid Peroxidation (MDA) Assay Kit (Sigma-Aldrich, # MAK568) following the manufacturer’s protocol using 12 µmol/L CDIM compounds and treatment time of 16 h. The MDA content was calculated for individual treatment groups from the standard curve.

### Western blotting and quantitative real-time polymerase chain reaction (qPCR) assay

Cells were treated with vehicle (DMSO) and ligands (DIM-3,5 compounds and Ferrostatin-1), whole cell lysates were analyzed by Western blots as described [18–20]. Antibodies used for protein detection are listed in the Supplementary Table 1. ImageJ software was used to quantify the Western blots. For determining CD71 (TFRC) mRNA, cells were treated with DIM-3,5 compounds, total RNA content was extracted using the RNeasy Mini Kit (QIAGEN, 74104), and qPCR was performed using the amfiSure qGreen Q-PCR Master Mix (GenDEPOT) as described [55]. The β-Actin primer sequences forward: 5′-ATCGGTTGGTGCCACTGAATGG-3′ and reverse: 5′-AGGTCTTTGCGGATGTCCACGT-3′ were used as a reference gene to normalize the input cDNA and the primer sets for the CD71 were forward: 5′- ATCGGTTGGTGCCACTGAATGG-3′ and reverse 5′- ACAACAGTGGGCTGGCAGAAAC-3′.

### Small RNA interference (siRNA) and plasmid transfection and luciferase assays

Cells were seeded at a density of 1.5 × 10^5^ cells per well on a 6-well plate. Lipofectamine RNAiMAX (Invitrogen, # 56531) was used for the transfection and carried out according to the manufacturer’s protocol with the mixture of Gibco-Opti-MEM (Fisher Scientific) and oligos. After 72 h, cells were harvested and lysed with RIPA buffer for protein analysis. The siRNAs that were used for this study are siNR4A1 (SASI_Mm01_00077215- NR4A1_C, SASI_Mm01_00077216- NR4A1_D), siNR4A2 (SASI_Hs02_0034_1056-NR4A2-A, SASI_Hs02_0034_1057-NR4A2-B), siSp1 (SASI_Hs02_00333289-Sp1-A, SASI_Hs02_00070994-Sp1-B), siSp3 (SASI_Hs01_00211941-Sp3-A, SASI_Hs01_00211941 = -Sp3-C), siSp4 (SASI_HS01-00114420-Sp4-A, SASI_HS01-00114421-Sp4-B) and scrambled siRNA (CGU ACG CGG AAU ACU UCG A (Sigma-Aldrich). The plasmid containing Sp1 coding sequence (Addgene plasmid # 12097) and empty vector as negative control (Addgene plasmid # 20783) using Lipofectamine™ 3000 reagent (Invitrogen, # L3000008) according to the manufacturer’s instructions. After 48 h, cells were harvested and lysed with RIPA buffer for protein analysis by Western blot. The CD71 (TFRC) promoter construct CD71-(luc) contained a −2000 to +200 CD71 promoter insert that was purchased from VectorBuilder (VectorBuilder Inc., VB240731-1429ezh). The experiment was carried out following the manufacturer’s protocol and as previously described [53].

### Chromatin immunoprecipitation (ChIP) assay

The ChIP assay was conducted using the ChIP-IT Express kit (Active Motif, # 53008), following the manufacturer’s protocol and primer sets for this gene (Supplementary Table 2). Cells were treated with DIM-3,5 compounds at a concentration of 10 µmol/L, along with vehicle control (DMSO), and binding to the CD71 promoter using qPCR was determined as described [53].

## Statistical analysis

Statistical analysis was performed using a t-test. All the in vitro experiments were carried out in triplicate and no less than three independent experiments. Data are expressed as the mean ± SD. One-way analysis of variance (Dunnett’s) was used to determine statistical significance, and *P* < 0.05 were considered statistically significant. Detailed methods are provided in the Supplementary files.

## Supplementary information

Supplementary Material

Original Western Blots

## Data Availability

The datasets used and/or analyzed during the current study are available from the corresponding author on reasonable request.
