# Supplementary material for: Orphan nuclear receptor 4A1 (NR4A1) and NR4A2 are endogenous regulators of CD71 and their ligands induce ferroptosis in breast cancer
Source: Cell Death Dis. 2025 Nov 3;16(1):776. doi: 10.1038/s41419-025-08143-5 (PMC12583510; doi:10.1038/s41419-025-08143-5)
Supplement: Supplementary file 1 — Supplementary Material [file 41419_2025_8143_MOESM1_ESM.docx]

**SUPPLEMENTARY MATERIAL**

Manuscript Title: **ORPHAN NUCLEAR RECEPTOR 4A1 (NR4A1) AND NR4A2 ARE ENDOGENOUS REGULATORS OF CD71 AND THEIR LIGANDS INDUCE FERROPTOSIS IN BREAST CANCER**

Arafat Rahman Oany^1^, Srijana Upadhyay^1^, Wai Ning Tiffany Tsui^1^, Amanuel Hailemariam^1^, Sarah Latka^2^, John Landua^5^, Sandra D. Scherer^3^, Alana L. Welm^3^, Hugo Villanueva^2,4^, Michael T. Lewis^2,5^ and Stephen Safe^1^*.

^1^Department of Veterinary Physiology and Pharmacology, College of Veterinary Medicine, Texas A&M University, College Station, TX 77843 USA

^2^Advanced Technology Cores, Baylor College of Medicine, Houston, TX 77030 USA

^3^Department of Oncological Sciences, Huntsman Cancer Institute, University of Utah, Salt Lake City, UT 84112 USA

^4^Otolaryngology – Head and Neck Surgery, Baylor College of Medicine, Houston, TX 77030 USA

^5^Breast Center, Baylor College of Medicine, Houston, TX 77030 USA

**Table of Content**

Cover Page……………………………………………………………………………………………………Pg 1

Table of Content………………………………………………………………………………………………Pg 1

Supplementary Table 1………………………………………………………………………………………Pg 2

Supplementary Table 2………………………………………………………………………………………Pg 3

Supplementary Figure 01……………………………………………………………………………………Pg 3

Supplementary MATERIALS AND METHODS……………………………………………………………Pg 3

**Supplementary Table1**

| **Antibody** | **Source** | **Catalog No./Identifier** | **WB Dilution** |
| --- | --- | --- | --- |
| GAPDH (D16H11) XP® Rabbit mAb | Cell Signaling | 5174 | 1:1000 |
| EGFR | Santa Cruz Biotechnology | c-373746 | 1:500 |
| Integrin β1 (D2E5) Rabbit mAb | Cell Signaling | 9699 | 1:1000 |
| c-Myc | Cell Signaling | 9402 | 1:500 |
| Cleaved PARP (Asp214) | Cell Signaling | 9541 | 1:1000 |
| PARP (46D11) | Cell Signaling | 9532 | 1:1000 |
| Caspase-3 | Cell Signaling | 9662 | 1:1000 |
| Cleaved Caspase-3 (Asp175) | Cell Signaling | (5A1E) | 1:1000 |
| GPX4 (E5Y8K) Rabbit mAb | Cell Signaling | 59735 | 1:500 |
| xCT/SLC7A11 (D2M7A) Rabbit mAb | Cell Signaling | 12691 | 1:500 |
| CD71/TFRC (H68.4) | Santa Cruz Biotechnology | sc-65882 | 1:1000 |
| β-Actin | Sigma | A5316 | 1:1000 |
| NR4A1 | Abcam | Ab283264 | 1:500 |
| NR4A2 | Santa Cruz Biotechnology | sc-376984 | 1:1000 |
| NR4A3 (NOR-1) (H7) | Santa Cruz Biotechnology | sc-393902 | 1:1000 |
| Sp1 (both ChIP and western) | Santa Cruz Biotechnology | Sc-17824 | 1:500 |
| Sp3 | Santa Cruz Biotechnology | Sc-644 | 1:500 |
| Sp4 (both ChIP and western) | Santa Cruz Biotechnology | Sc-390124 | 1:500 |
| Bcl-2 (D17C4) Rabbit mAb | Cell Signaling | 3498 | 1:500 |
| NR4A1 (for ChIP) | Santa Cruz | Sc-365113 X | N/A |
| Mouse IgG (for ChIP) | Santa Cruz | Sc-2025 | N/A |

**Supplementary Table2**

| **Primer** | **Sequence** |
| --- | --- |
| TFRC (CD71) (Binding Site 1) | Forward: 5’ GAGCCCAGGAGTTCAAGACTA 3’  Reverse: 5’ ATTCCTGACCTCAGGTGATCT 3’ |
| TFRC (CD71) (Binding Site 2) | Forward: 5’ TACGTGCCTCAGGAAGTGAC 3’  Reverse: 5’ AGTGGCAGAAACAGTGGATG 3’ |
| TFRC (CD71) (Binding Site 3) | Forward: 5’ GTACGTGCCTCAGGAAGTGA 3’  Reverse: 5’ GAAATGACAACGAGGGGATG 3’ |

**Supplementary Figure 01:**

**
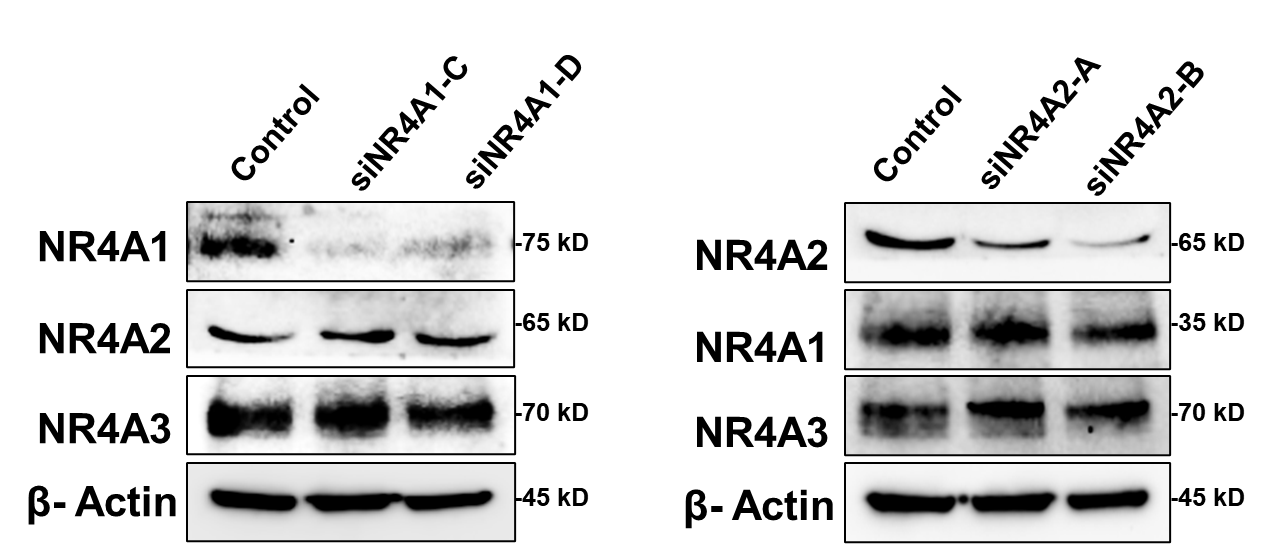
**

**Supplementary Figure 01: Effect of NR4A1 and NR4A2 Knockdown on the Expression of Other NR4A Family Members.** MDA-MB-231 cells were transfected with oligonucleotides targeting NR4A1 (A), NR4A2 (B) and after 72 hours, whole cell lysates were analyzed by Western blots as outlined in the methods.

**MATERIALS AND METHODS (Supplementary)**

**Cell culture, reagents, and ligands:**

MDA-MB-231 cells were cultured in Dulbecco’s modified Eagle’s medium (D-MEM) supplemented with 10% fetal bovine serum (FBS), and 100 µl (100x concentrated solution) per 10 mL of Gibco™ antibiotic-antimycotic solution (Fisher Scientific).MDA-MB-468 cells were cultured in D-MEM: Ham’s F-12 medium (50:50) supplemented with 10% FBS, and 100 µl (100x concentrated solution) per 10 mL of Gibco™ antibiotic-antimycotic solution (Fisher Scientific).4T1 cells were maintained in RPMI supplemented with 10% FBS, and 100 µl/10 ml of 100x Gibco™ antibiotic-antimycotic solution (Fisher Scientific). Typically, cells were grown in 60.8 cm^2^ culture plates with an air and CO_2_ ratio of (95:5) at 37ºC. Cells were checked for confluency and passaged about every 4 days. For the cell lysis, RIPA buffer (Thermo Scientific) and Luciferase Cell Culture Lysis 5X Reagent (Promega Corporation) were used. Xpert Protease Inhibitor Cocktail Solution (100X) (GenDEPOT) and Xpert Phosphatase Inhibitor Cocktail Solution(100X) (GenDEPOT) were also used for cell lysis. 1,1-bis(3′-indolyl)-1-(3,5-disubstitutedphenyl)methane (DIM-3,5) analogs containing 3,5-dichlorophenyl (DIM-3,5-Cl_2_) and 3-chloro-5-trifluoromethylphenyl (DIM-3-Cl-5-CF_3_) were used in this study as ligands [53]. Ferrostatin-1 (Fer-1) (was purchased from Selleck Chemicals LLC (# S7243). Less than 0.15% by volume of the solvent dimethyl sulfoxide (DMSO) was typically added to the medium as the control since compounds were added in the DMSO. Three replicates’ studies were conducted for every treatment group.

**Cell proliferation assay**

Human and mouse breast cancer cells were seeded in a 96-well plate with appropriate growth media at 2 × 10^4^ cells per well for cell proliferation experiments. After incubation for 12 hours, cells were treated with several concentrations of bis-indole compounds (2.5, 5, 7, 10, 12, and 15 μmol/L) for 24 hours. For the cell viability studies the XTT assay was performed using the XTT Cell Viability Kit (Cell Signaling Biotechnology, # 9095) for 24 hours. The absorbance was measured on a plate reader (BioTek Cytation 5, Agilent) at 450 nm and the fraction of cell survival was assessed.

**Tissue slice and PDxO experiments**

Clinical information and individual demographics for all BCM PDX models can be found in the BCM PDX Portal (https://pdxportal.research.bcm.edu/). Tumors were allowed to grow to 1,000cm3, harvested, and then cored using a 5mm diameter coring press. The resulting tumor cylindrical core was transferred to an Alabama Research and Development Tissue Slicer and sliced into ~400µm thick discs according to the manufacturer’s guidelines. Media (Advanced DMEM/F12 with 5% FBS, 10mM HEPES, 1× Glutamax, 1μg ml–1 hydrocortisone, 50μg ml–1 gentamicin, 10ng ml–1 hEGF and 10 μM Y-27632) used to grow the tumor disc with or without DIM-3-Cl-5-CF_3_ or DIM-3,5-Cl_2_, sealed with a gas permeable membrane and incubated with gentle agitation at 37 ºC with 18% O_2_ / 5% CO_2_. After 72 hours, the tumor discs were rinsed with PBS, snap frozen and stored at -80 ºC.

Tumor organoids, briefly, cryopreserved PDX tumor fragments were thawed and thoroughly washed in advanced DMEM/F12 medium prior to digestion in a GentleMACS tissue dissociator (Miltenyi biotec) according to manufacturer recommendations. Dissociated fragments were washed in organoid base medium and debris was removed using subsequent cell straining steps. Organoids were counted and seeded by embedding in Matrigel (Corning) and adding sufficient medium to submerge the dome completely.

Mature PDxOs (up to 100 um size) embedded in Matrigel were released from domes by incubation with dispase treatment at 37^o^C. PDxOs used for protein analysis were seeded in domes in 12-well plates at a density of 10,000 organoids per dome and were collected 24 hours after drug treatment with 15 uM DIM-3,5-Cl_2_ or DIM-3-Cl-5-CF_3_.

**Measurement of ROS**

Briefly, the human (MDA-MB-231 and MDA-MB-468) and mouse (4T1) breast cancer cells were cultured at a density of 3.0 × 10^5^ cells per well in 6-well plates with appropriate media containing 2.5% charcoal-stripped FBS for 24 hours. Cells were then treated with DIM-3,5-Cl_2_, and DIM-3-Cl-5-CF_3_ compounds at a concentration of 12 µmol/L, along with vehicle control (DMSO) for 16 hours, the media was separated and washed with phosphate-buffered saline (PBS) (2X). The CM-H_2_DCFDA probe was added into the pre-warmed Hanks' Balanced Salt Solution (HBSS) (Thermo Fisher) with a final concentration of 1 µmol/ L and incubated for 45 minutes. Finally, the loading buffer was removed and replaced with the appropriate growth media and observed under the fluorescence microscope (The EVOS FL Imaging System, Thermo Scientific) for the live cell fluorescence analysis (excitation and emission, in 495/517 nm) and detected by using a plate reader (BioTek Cytation 5, Agilent) for quantitation.

**Lipid peroxidation assay**

The experiment was carried out using the manufacturer’s protocol, and the breast cancer cells were cultured at a density of 3.0 × 10^5^ cells/ well in 6-well plates with appropriate media containing 2.5% charcoal-stripped FBS for 24 hours. Cells were treated with DIM-3,5-Cl_2_, and DIM-3-Cl-5-CF_3_ compounds at a concentration of 12 µmol/L, along with vehicle control (DMSO) for 16 hours and incubated with the probe for 30 minutes. Cells were washed with PBS (2X) and stained with a Hoechst dye (0.1 5 μg/mL solution). An inverted fluorescence microscope system (EVOS FL Imaging System, Thermo Scientific) was used to visualize the fluorescence signals. The images were analyzed and quantitated by ImageJ software. Three replicates of each control (DMSO) and treatment were used.

**Determination of malondialdehyde (MDA)**

Briefly, cells were seeded at a density of 3.0 × 10^5^ cells per well in 6-well plates for 24 hours with appropriate media. Cells were then treated with DIM-3,5-Cl_2_, and DIM-3-Cl-5-CF_3_ at a concentration of 12 µmol/L, along with vehicle control (DMSO) for 16 hours. Cells were then lysed with the MDA lysis buffer and centrifuged at 12,500 x g for 10 minutes and the supernatants were collected and incubated with thiobarbituric acid (TBA) at 95°C for 1 hour. MDA standards were prepared and the fluorescence absorbances were measured at λ_Ex_/λ_Em_ = 530/560 nm using a plate reader (BioTek Cytation 5, Agilent) for the sample and standard.

**Western blotting**

Cells were seeded at densities ranging from 1.0 to 3.0 × 10^5^ cells per well in 6-well plates for 24 hours with appropriate media for different experiments. After treatments, cells were lysed with RIPA buffer which was mixed with protease and phosphatase inhibitors and analyzed following the Bradford assay (Beckman DU 640). For most of the western blotting, equal amounts of protein were loaded on 10 percent SDS-polyacrylamide gels and transferred onto a PVDF membrane, (Thermo scientific) using Mini Trans-Blot Electrophoretic Transfer Cell (BioRad, #1703930). After blocking the membrane with 5% milk, protein bands were incubated with primary and secondary antibodies. Protein levels were analyzed by using ChemiDoc MP Imaging System (BioRad, # 12003154) for high-end imaging, and Immobilon Western Chemiluminescent HRP Substrate (Millipore Sigma) was used for detection.

**Quantitative real-time polymerase chain reaction (qPCR) assay**

For analysis of CD71 (TFRC) mRNA, cells were seeded at a density of 3.0 × 10^5^ cells per well in 6-well plates for 24 hours with appropriate media. Following treatment, total RNA content was extracted. To eliminate any contamination, the TURBO DNA-free Kit was used to treat the RNA (Invitrogen, # 3040986), and cDNA synthesis was conducted using the High-Capacity cDNA Reverse Transcription Kit (Applied Biosystems™, # 4374967). The qPCR amplifications were carried out using a Lightcycler 480 (Bio-Rad) according to the manufacturer’s protocols. Fold change calculation was performed using the 2^−ΔΔCt^ method.

**Small RNA interference (siRNA) and plasmid transfection:**

(Described in detail in the manuscript)

**Luciferase assay**

Briefly, cells were seeded on 12 or 24-well plates at a density of 7.5 × 10^4^ or 4 × 10^4^ cells per well for 24 hours for knockdown and treatment, respectively. Transfection of CD71-luc plasmid was carried out by Lipofectamine™ 3000 Transfection Reagent (Invitrogen, # L3000008), and cells were treated with DIM-3,5 compounds, Mithramycin (Cayman Chemical, #11434) and vehicle (DMSO). Cells were harvested, and the lysates were extracted using Cell Culture Lysis 5X Buffer (Promega), and protein concentrations were determined through Bradford assay. The Luciferase Activity Reagent (Promega) was used to measure the luciferase activity of equal amounts of protein using the plate reader (BioTek Cytation 5, Agilent). Protein concentrations were used for the normalization of each sample.

**Chromatin immunoprecipitation (ChIP) assay**

Briefly, the cells were seeded on 143 cm^2^ culture plates with a density of 3 × 10^6^ cells per plate and grown for 24 hours. Cells were treated with DIM-3,5 compounds at a concentration of 10 µmol/L, along with vehicle control (DMSO), and incubated for 6 hours. Cells were crosslinked for 10 minutes at room temperature using a 1% formaldehyde solution; after cross-linking, the cells were scraped and collected into a pellet after quenching the excess formaldehyde with glycine. The pellets were sonicated in lysis buffer on ice at 50–70% intensity (5X) to get the desired sheared DNA. The sonicated chromatin was immunoprecipitated overnight at 4°C with rotation using protein G-conjugated magnetic beads and incubated with specific ChIP grade antibodies (IgG, Sp1, Sp4, NR4A1, and NR4A2) mentioned in the Supplementary Table 1. After the elution, the reverse crosslinking was performed, and the DNA was cleaned up using the Chromatin IP DNA Purification Kit (Active Motif, # 58002). The qPCR was performed using amfiSure qGreen Q-PCR Master Mix (GenDEPOT). The JASPAR database (<http://jaspar.genereg.net/>) was utilized to identify probable binding sites of Sp within the TFRC (CD71) promoter.
