# Supplementary material for: Orphan nuclear receptor 4A1 (NR4A1) and NR4A2 are endogenous regulators of CD71 and their ligands induce ferroptosis in breast cancer
Source: Cell Death Dis. 2025 Nov 3;16(1):776. doi: 10.1038/s41419-025-08143-5 (PMC12583510; doi:10.1038/s41419-025-08143-5)

Figure 1C

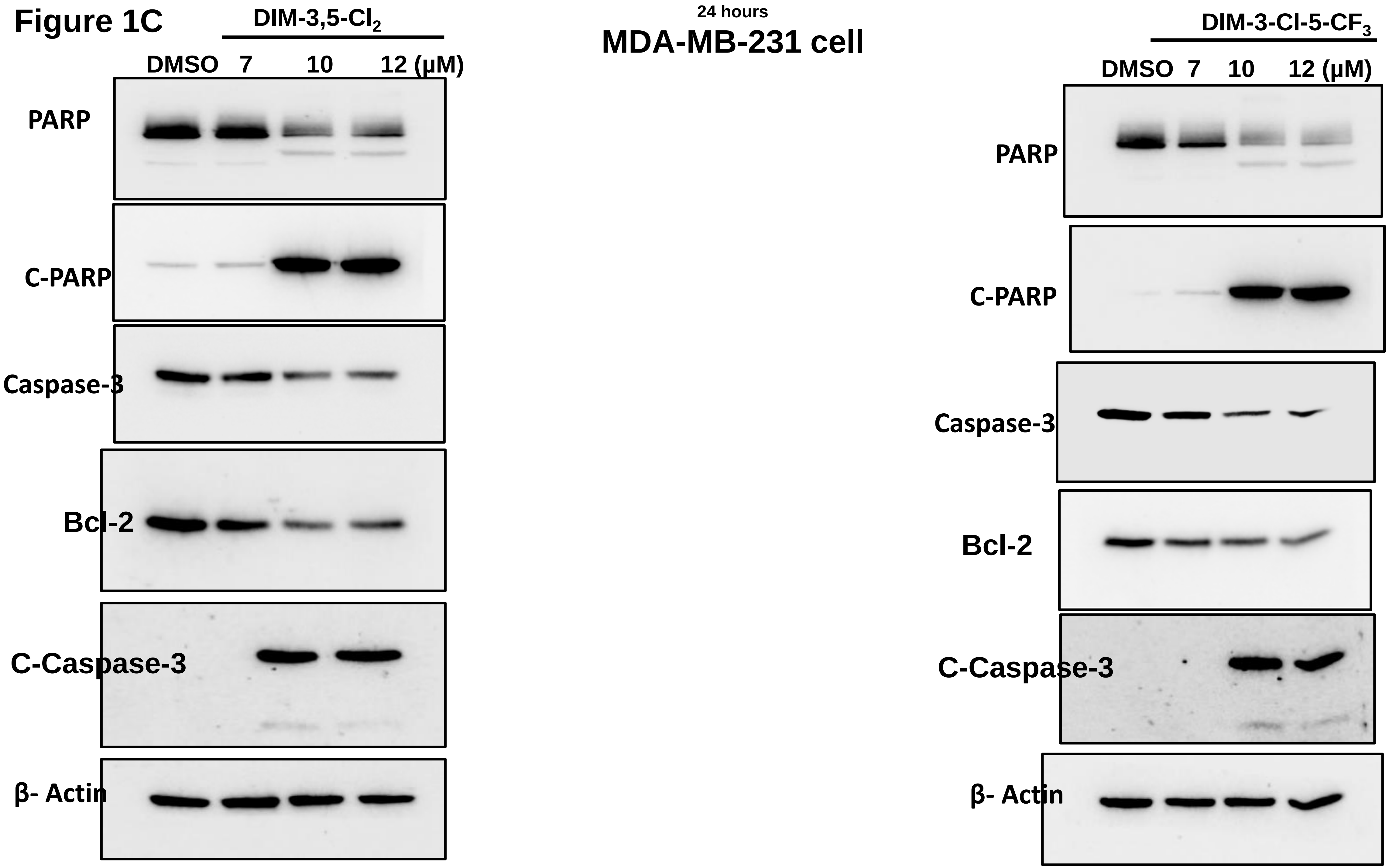

24 hours
MDA-MB-231 cell
DIM-3,5-Cl$_2$
DMSO 7 10 12 (μM)
PARP
C-PARP
Caspase-3
Bcl-2
C-Caspase-3
β- Actin
DIM-3-Cl-5-CF$_3$
DMSO 7 10 12 (μM)
PARP
C-PARP
Caspase-3
Bcl-2
C-Caspase-3
β- Actin

Figure 1C

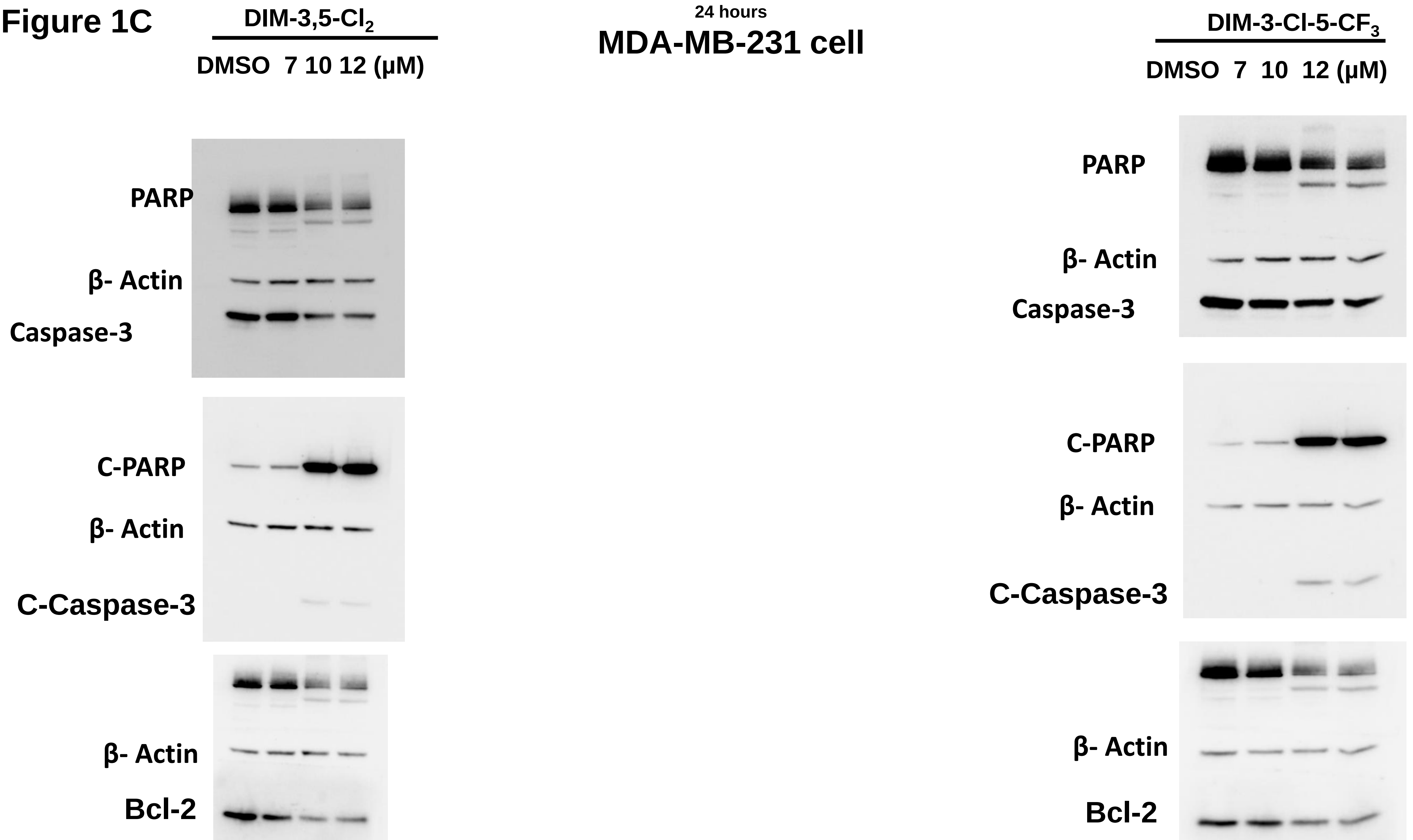

Full blot

Figure 1C

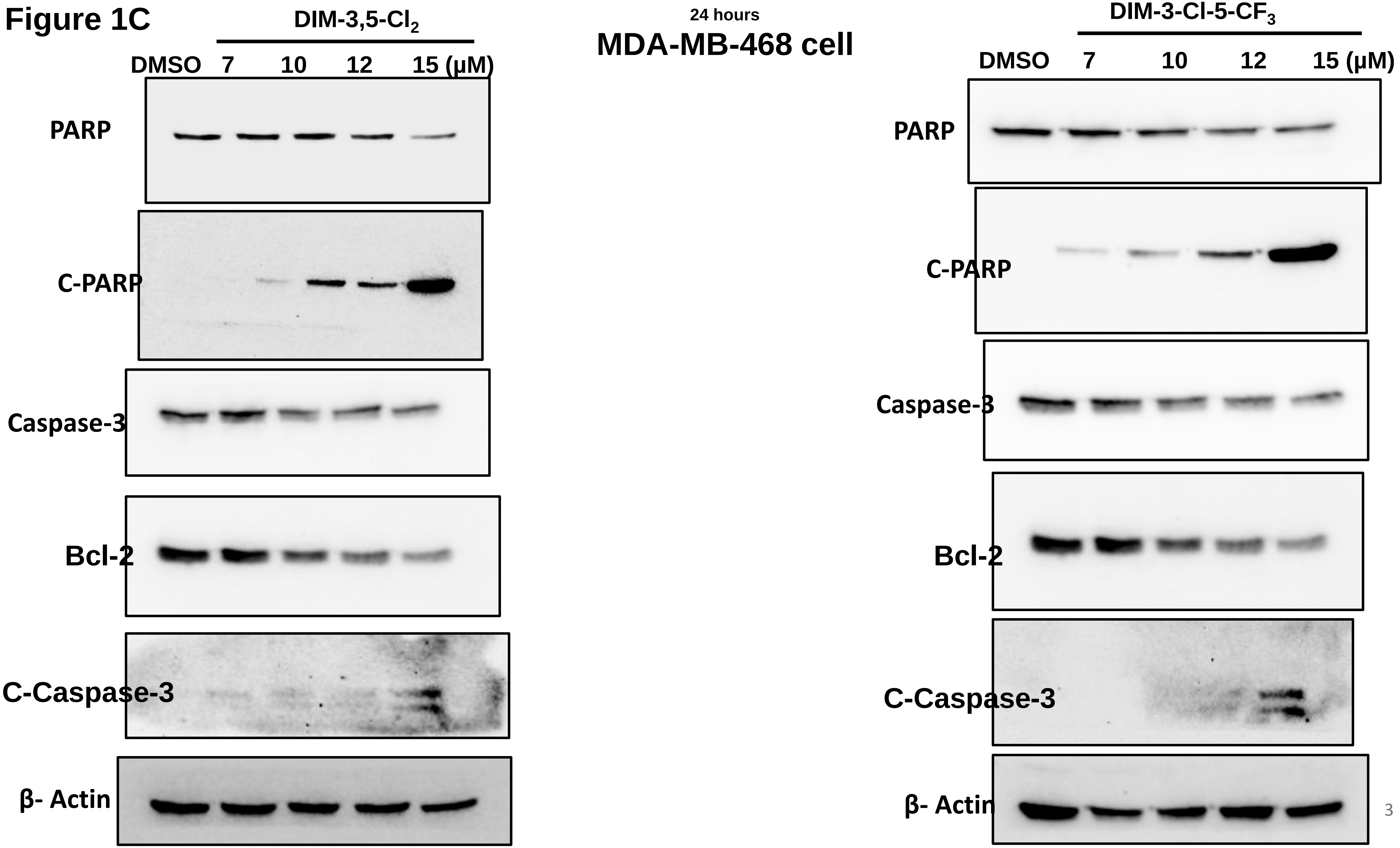

24 hours
MDA-MB-468 cell
DIM-3,5-Cl2
DMSO
7
10
12
15 (μM)
PARP
C-PARP
Caspase-3
Bcl-2
C-Caspase-3
β- Actin
DIM-3-Cl-5-CF3
DMSO
7
10
12
15 (μM)
PARP
C-PARP
Caspase-3
Bcl-2
C-Caspase-3
β- Actin

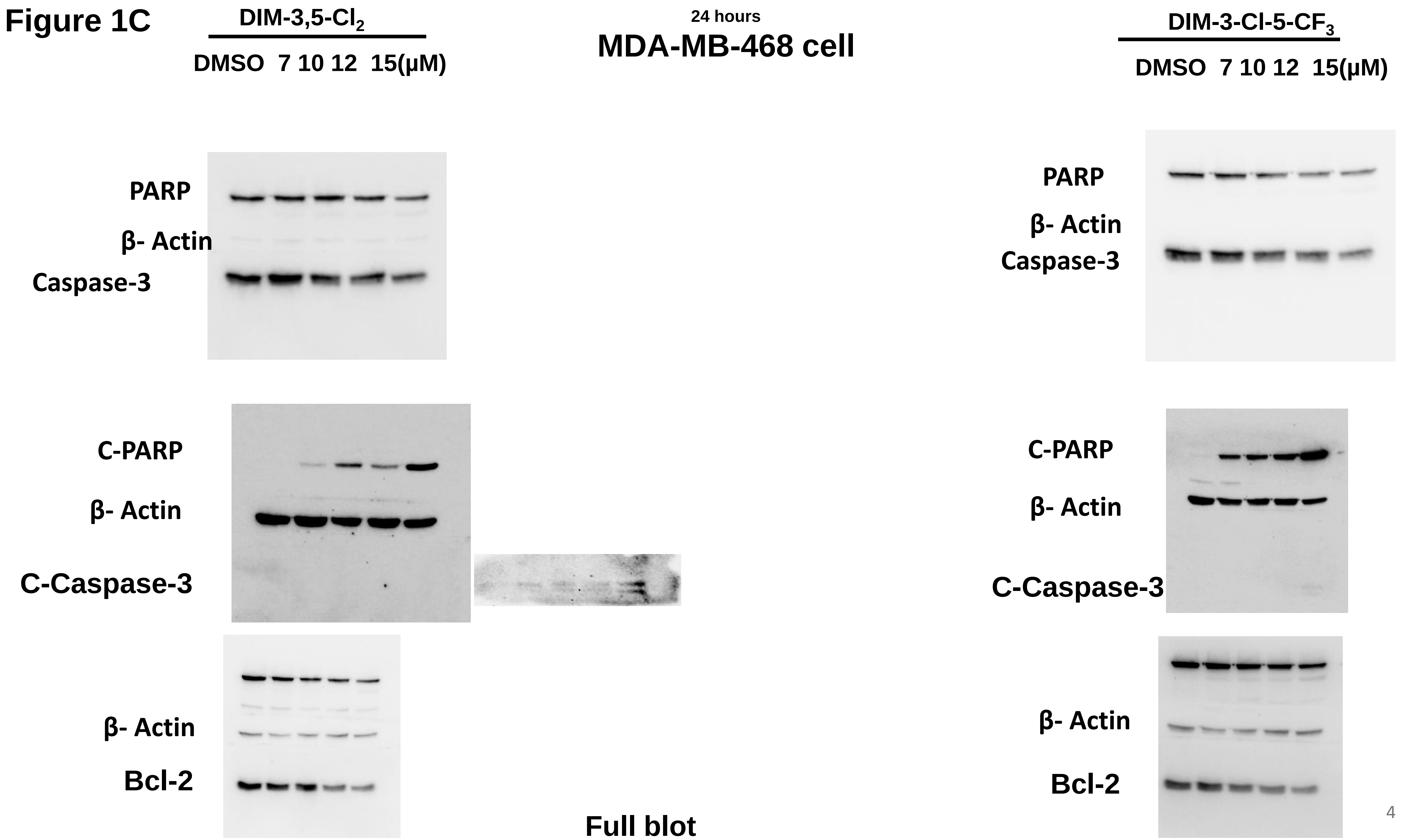
Figure 1C
DIM-3,5-Cl$_2$
24 hours
MDA-MB-468 cell
DIM-3-Cl-5-CF$_3$
DMSO 7 10 12 15(μM)
DMSO 7 10 12 15(μM)
PARP
β- Actin
Caspase-3
PARP
β- Actin
Caspase-3
C-PARP
β- Actin
C-Caspase-3
C-PARP
β- Actin
C-Caspase-3
β- Actin
Bcl-2
β- Actin
Bcl-2
Full blot

Figure 1C

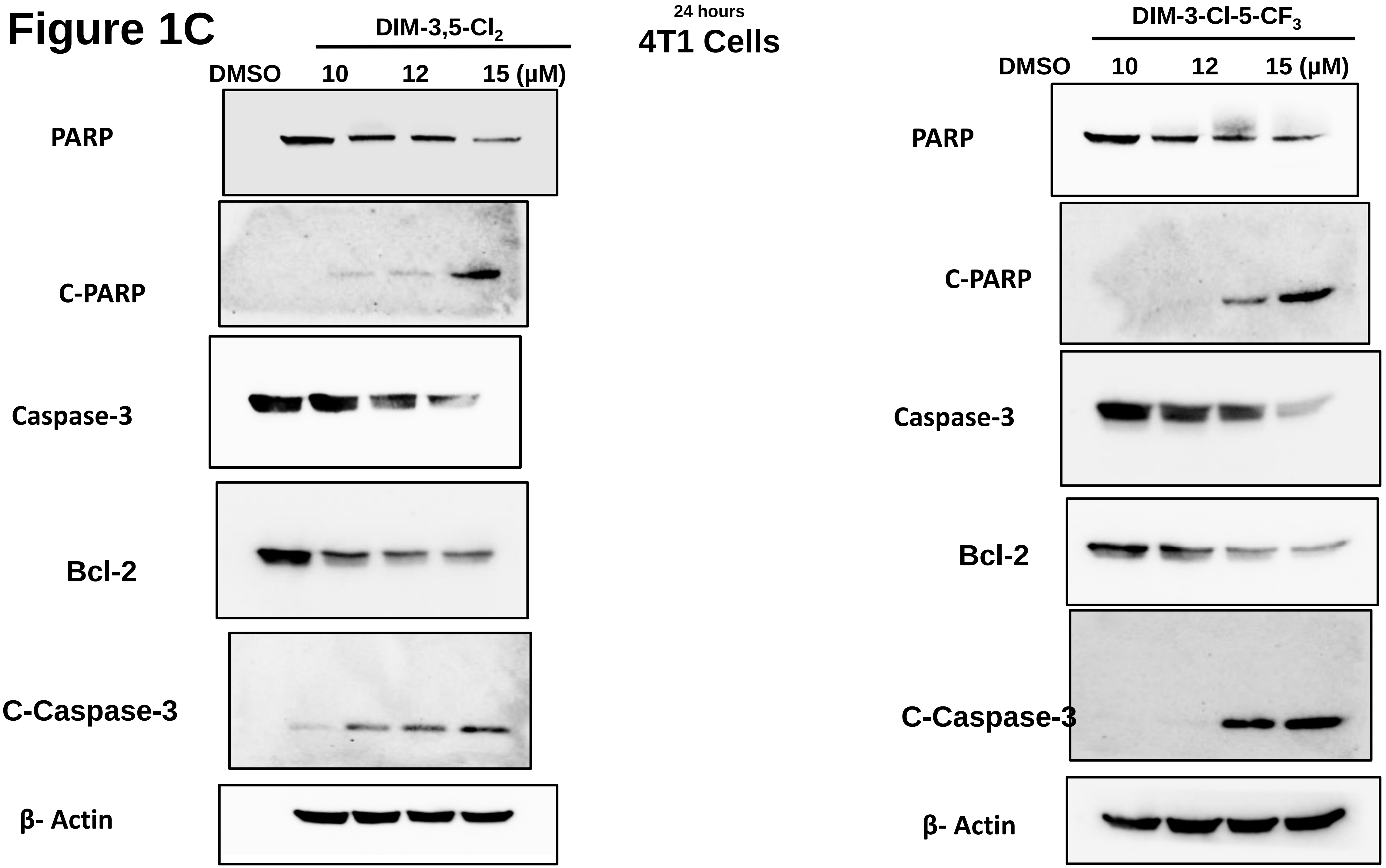
24 hours
4T1 Cells
DIM-3,5-Cl2
DMSO
10
12
15 (µM)
PARP
C-PARP
Caspase-3
Bcl-2
C-Caspase-3
β- Actin
DIM-3-Cl-5-CF3
DMSO
10
12
15 (µM)
PARP
C-PARP
Caspase-3
Bcl-2
C-Caspase-3
β- Actin

Figure 1C

24 hours
4T1 Cells

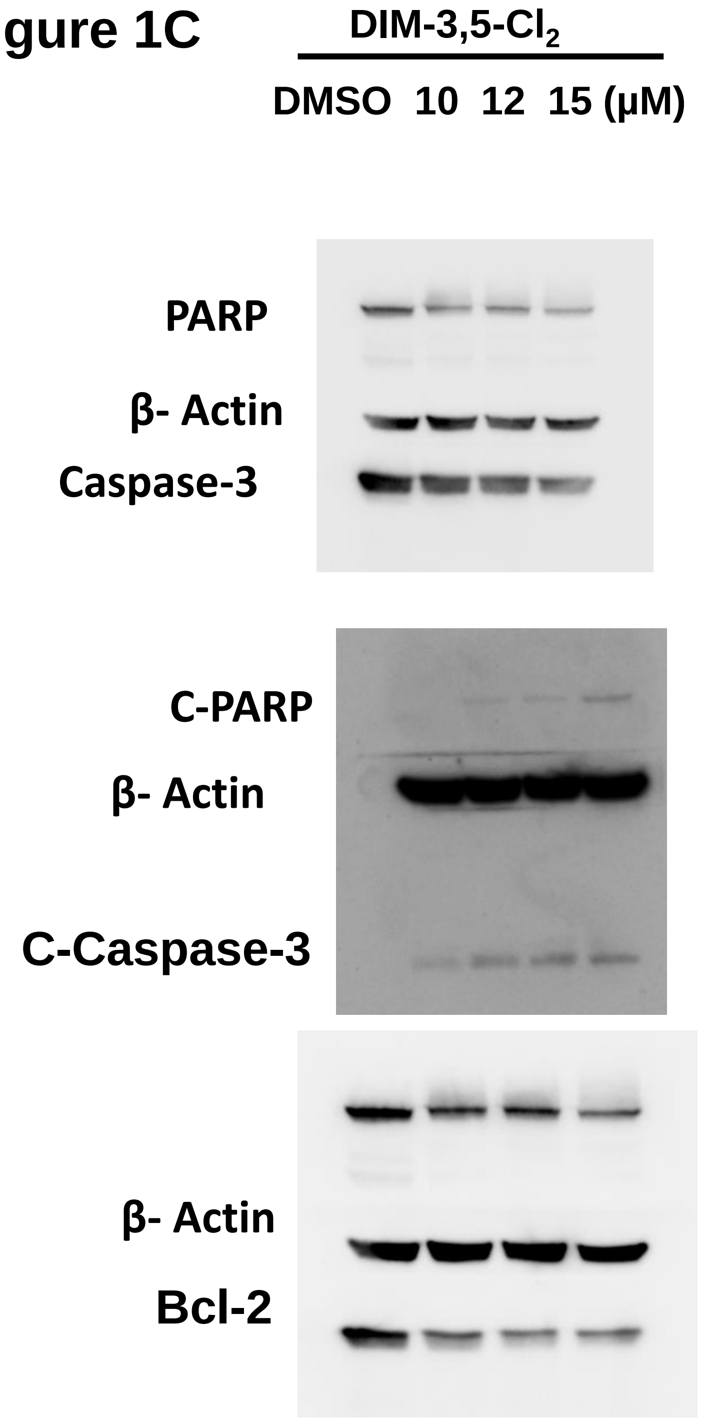

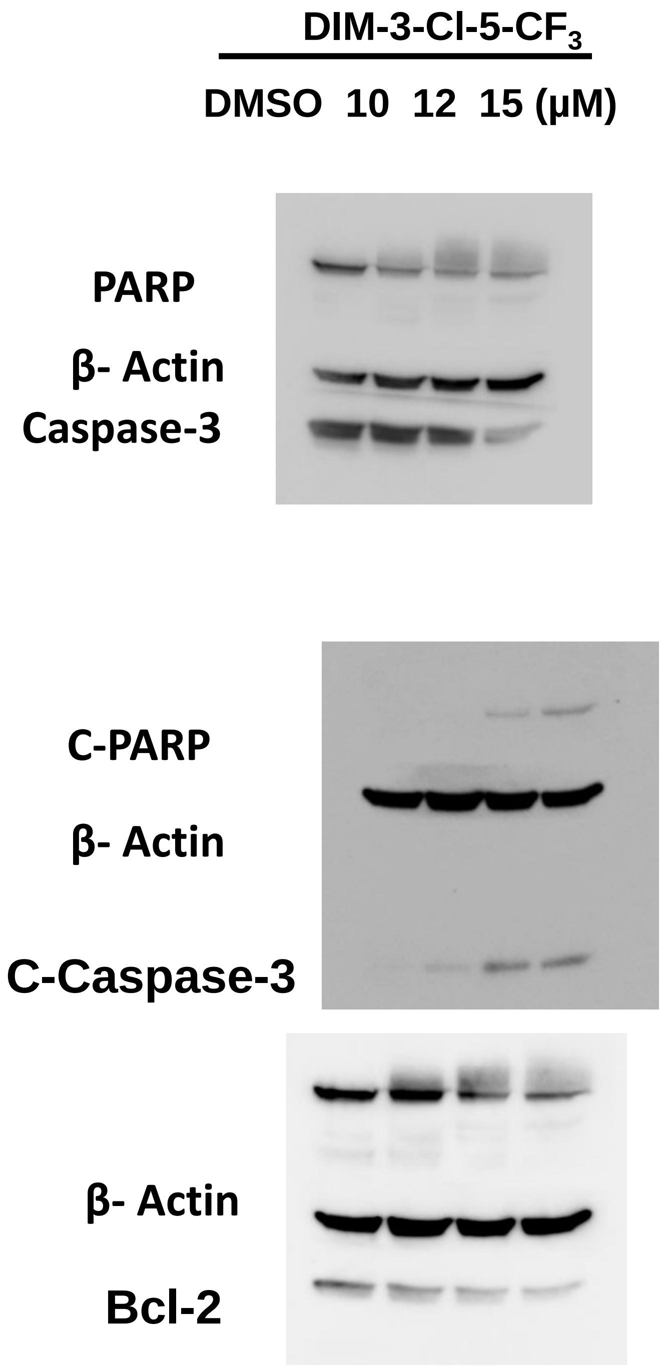

Full blot

Figure 1D

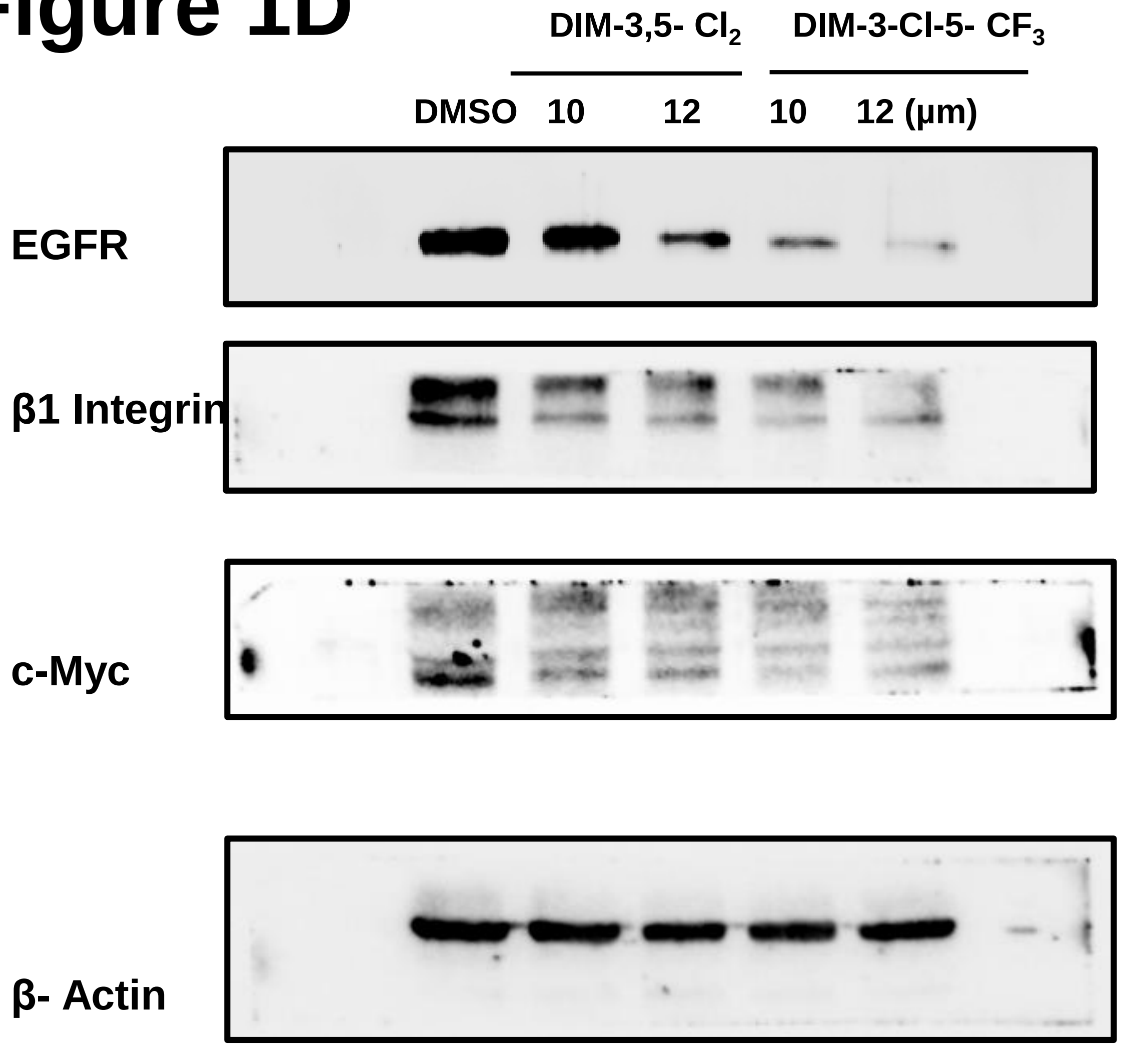

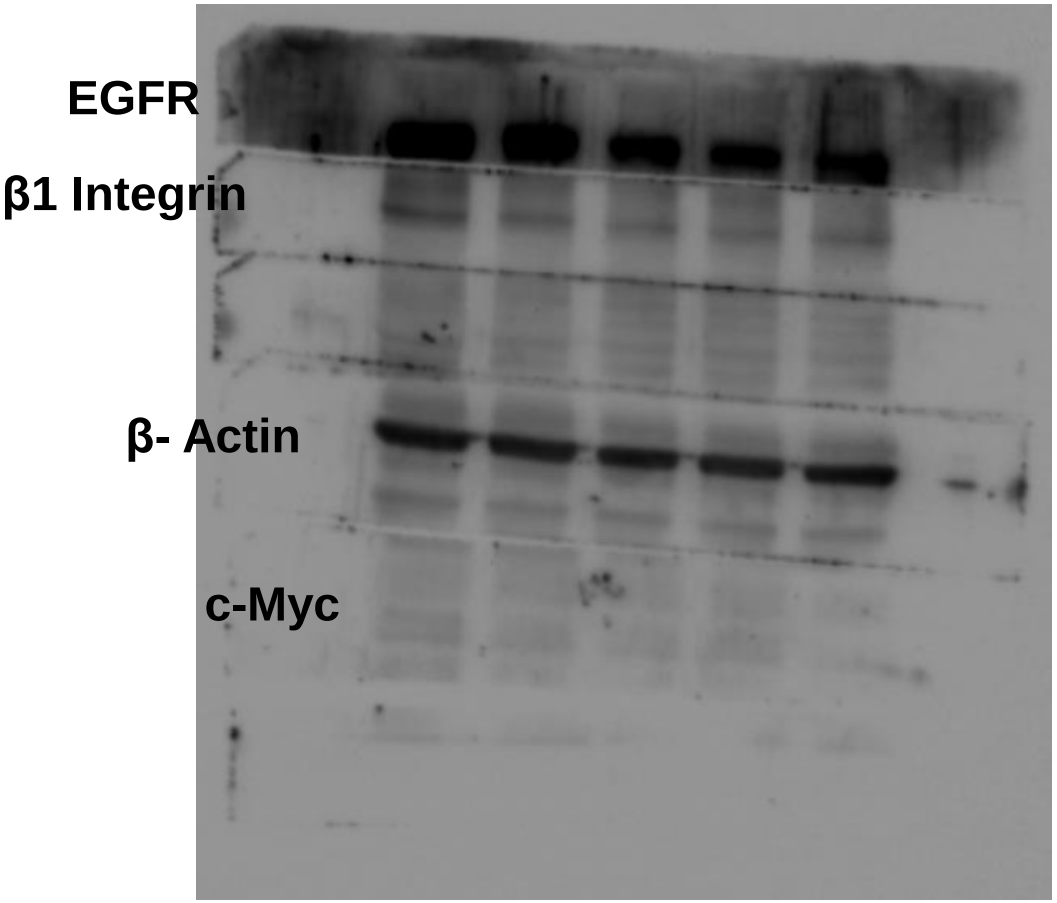

Full blot

Figure 1E

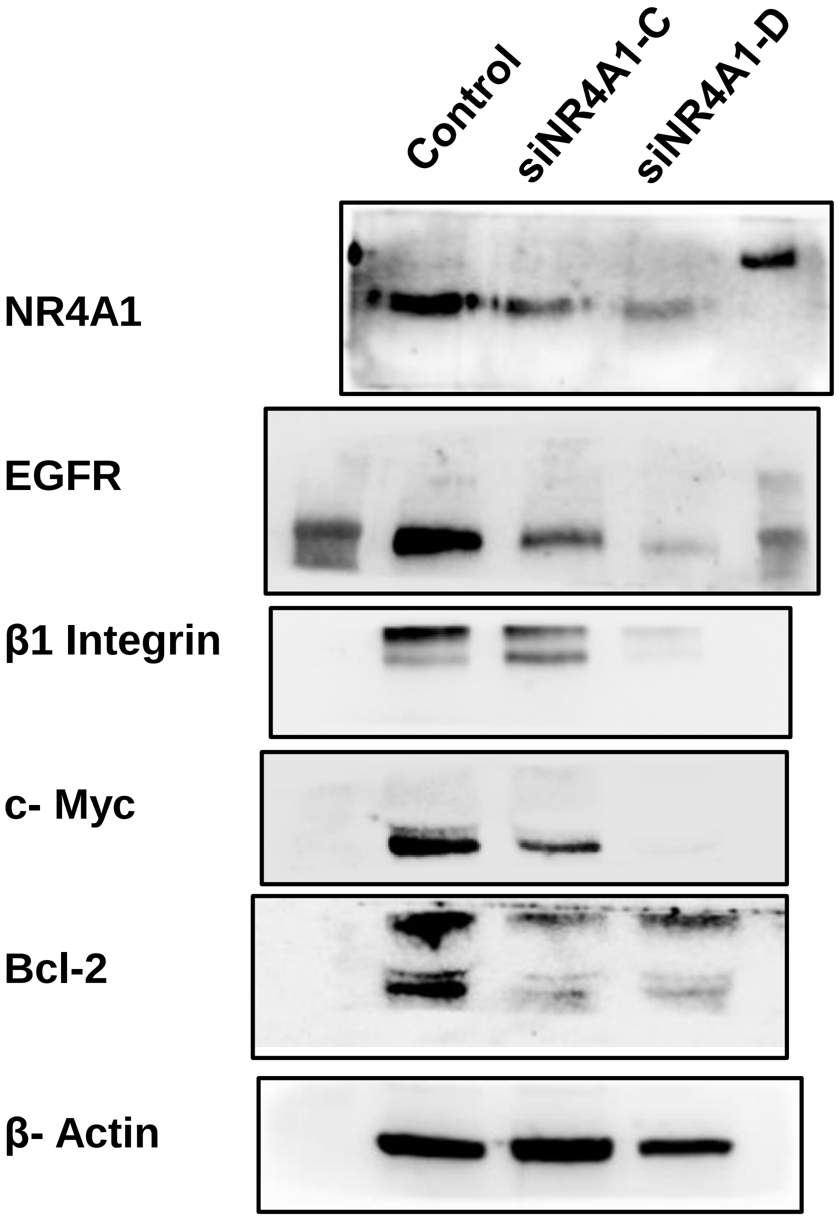

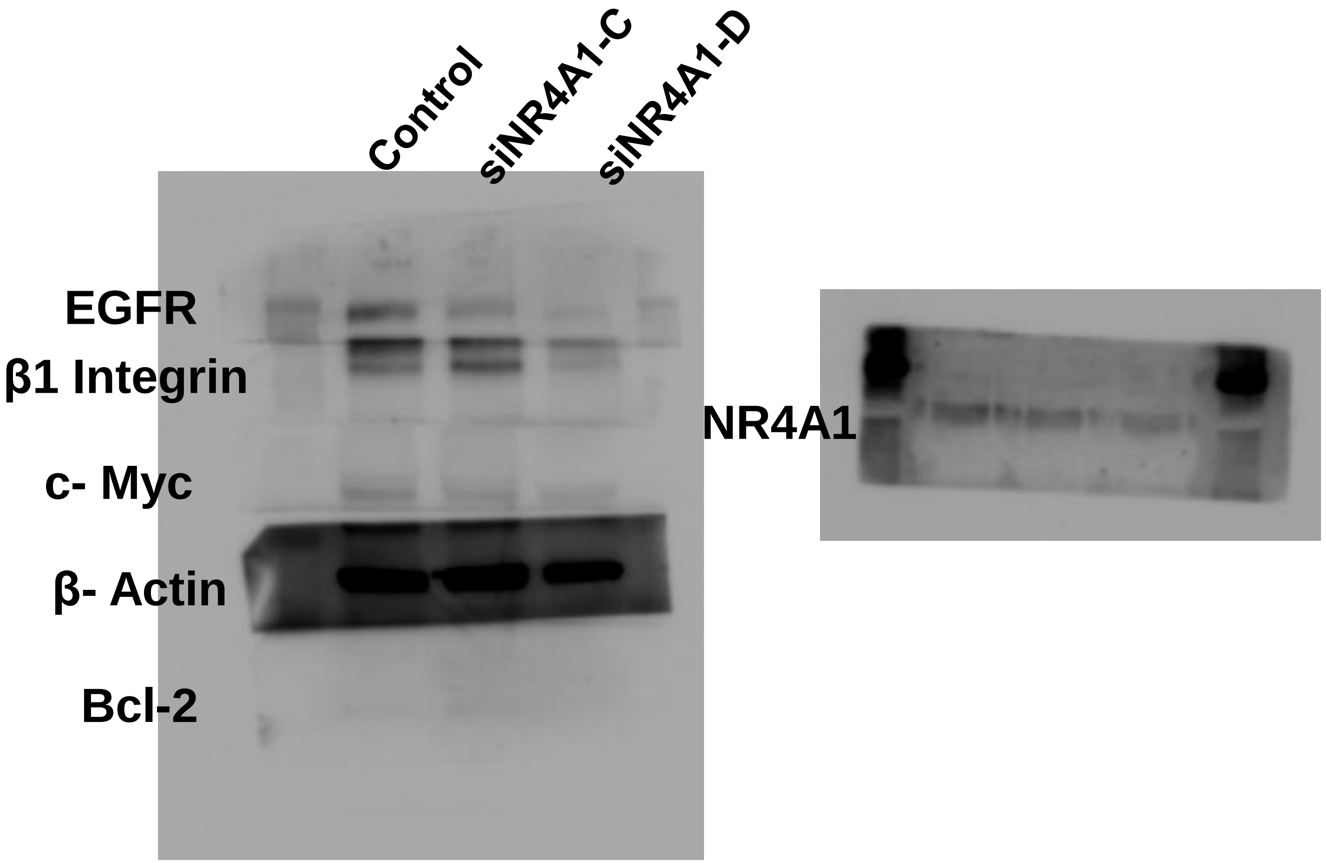

Full blot

Figure 1E

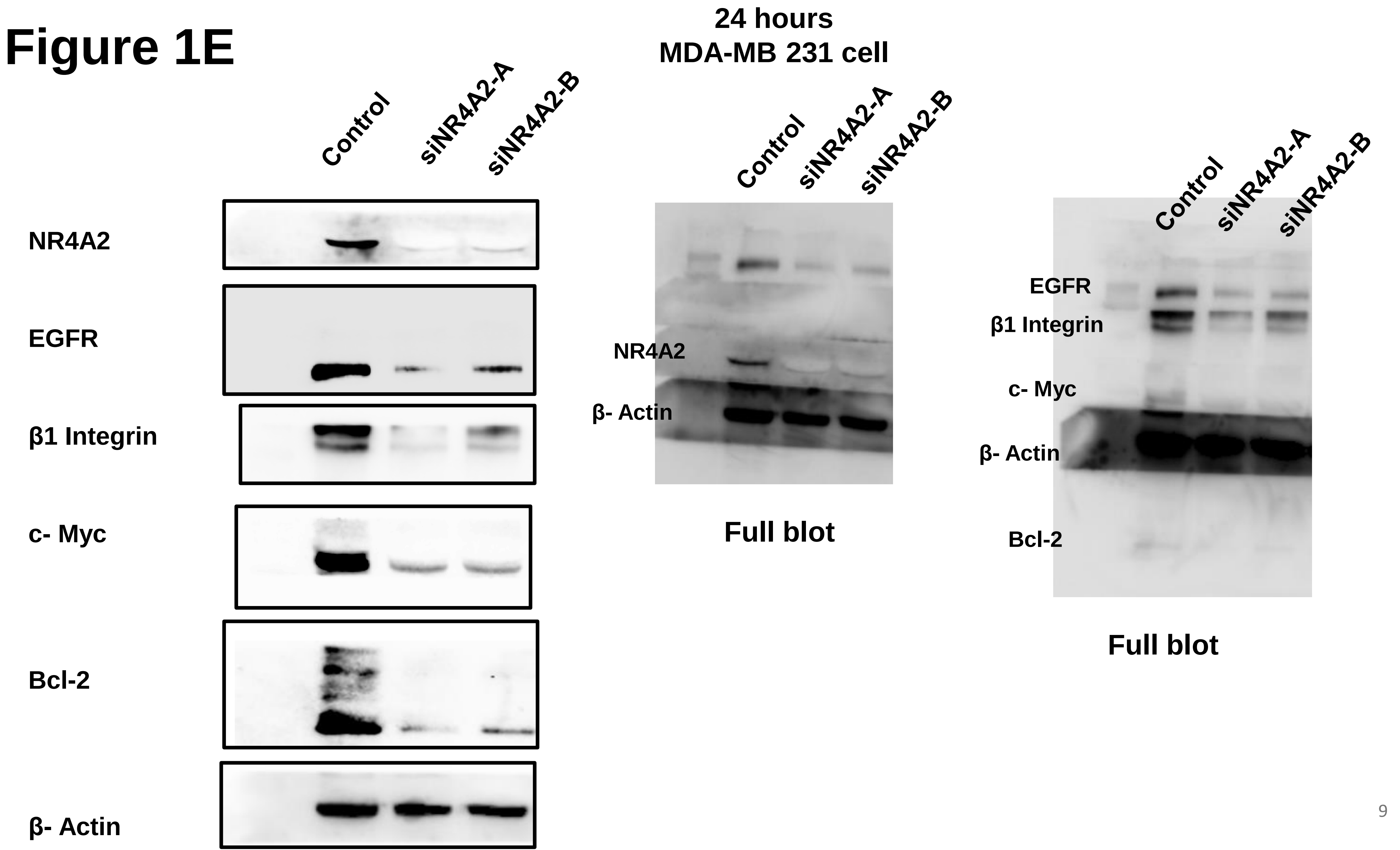
24 hours
MDA-MB 231 cell
Control
siNR4A2-A
siNR4A2-B
NR4A2
EGFR
β1 Integrin
c- Myc
Bcl-2
β- Actin
Control
siNR4A2-A
siNR4A2-B
NR4A2
β- Actin
Full blot
Control
siNR4A2-A
siNR4A2-B
EGFR
β1 Integrin
c- Myc
β- Actin
Bcl-2
Full blot

Figure 3A

24 hours
MDA-MB 231 cell

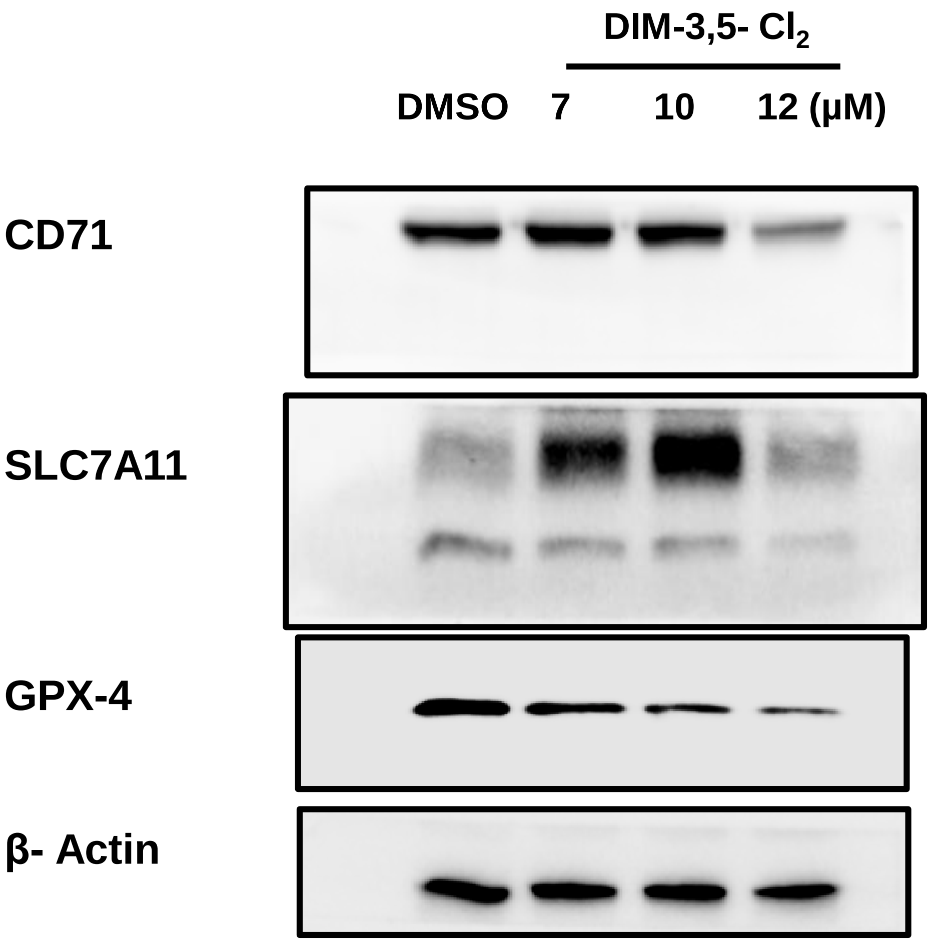

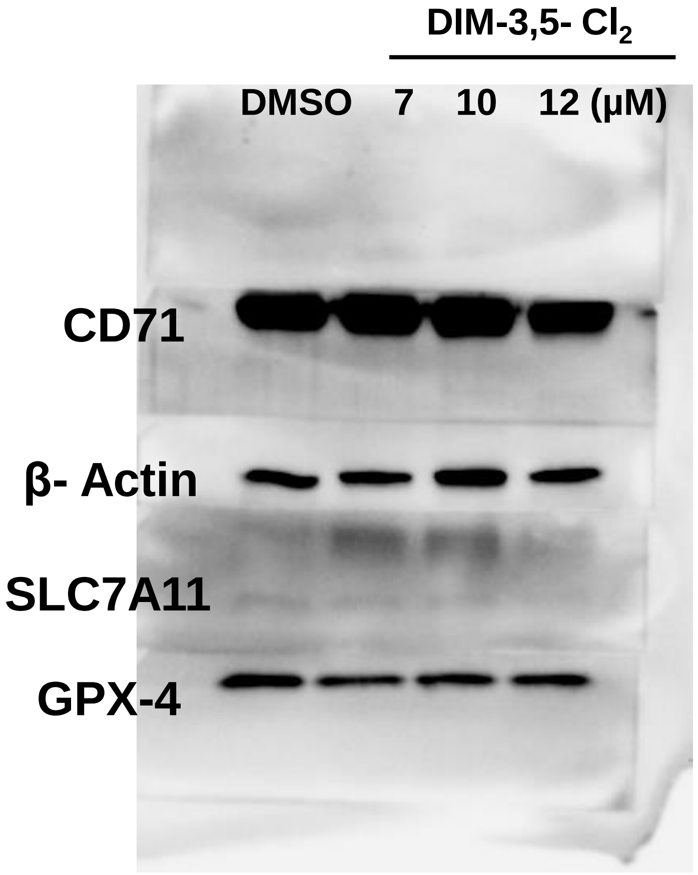

Full blot

Figure 3A

24 hours
MDA-MB 231 cell

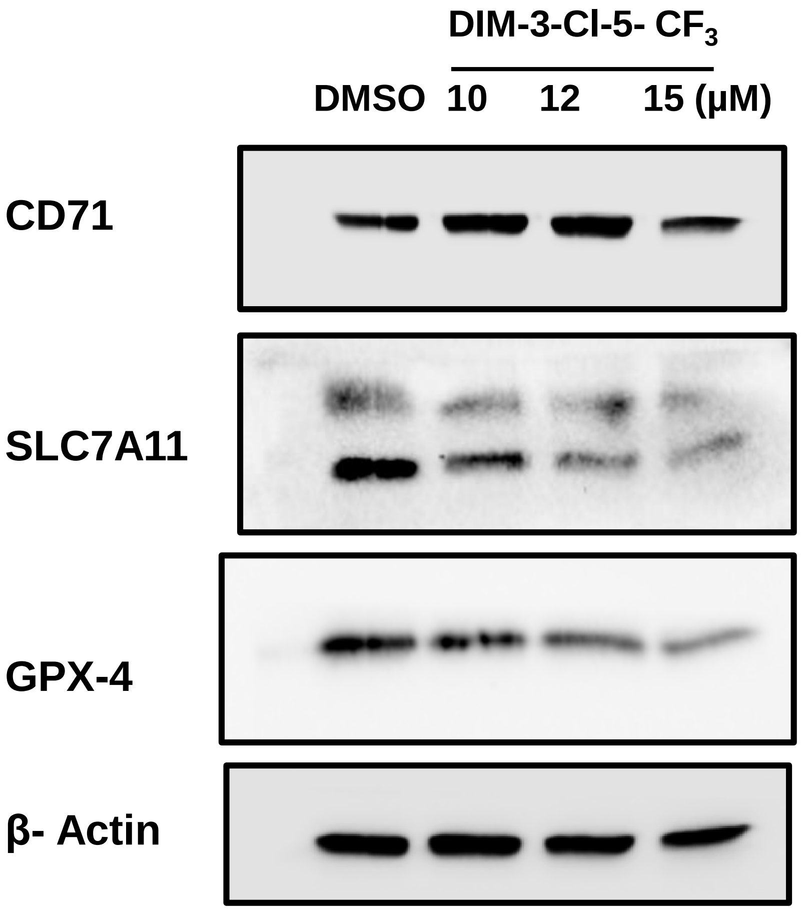

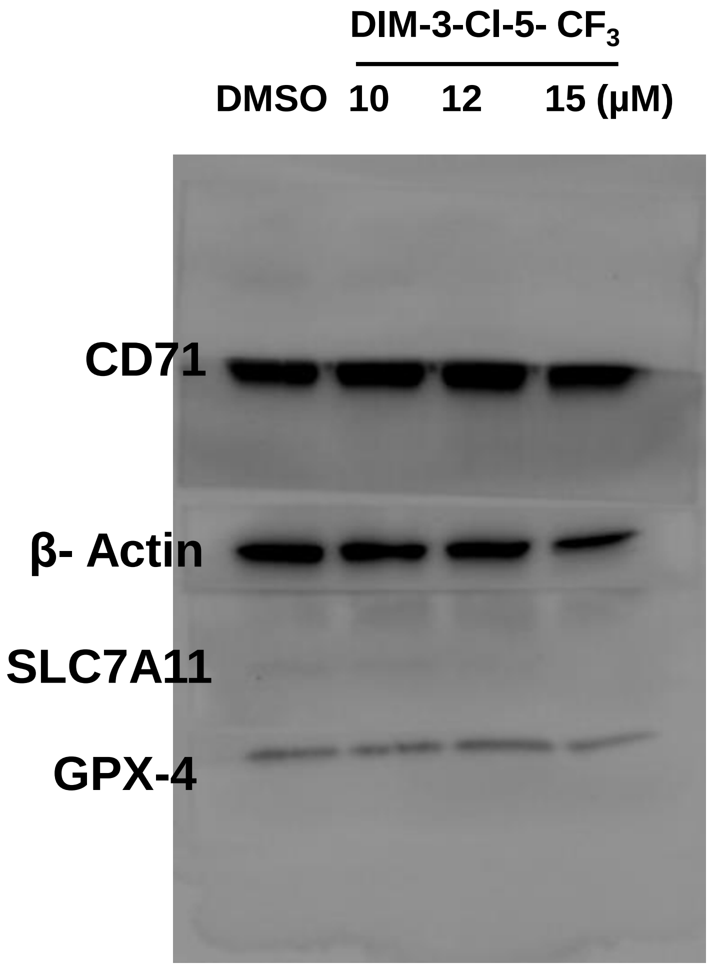

Full blot

Figure 3B

24 hours
MDA-MB 468 cell

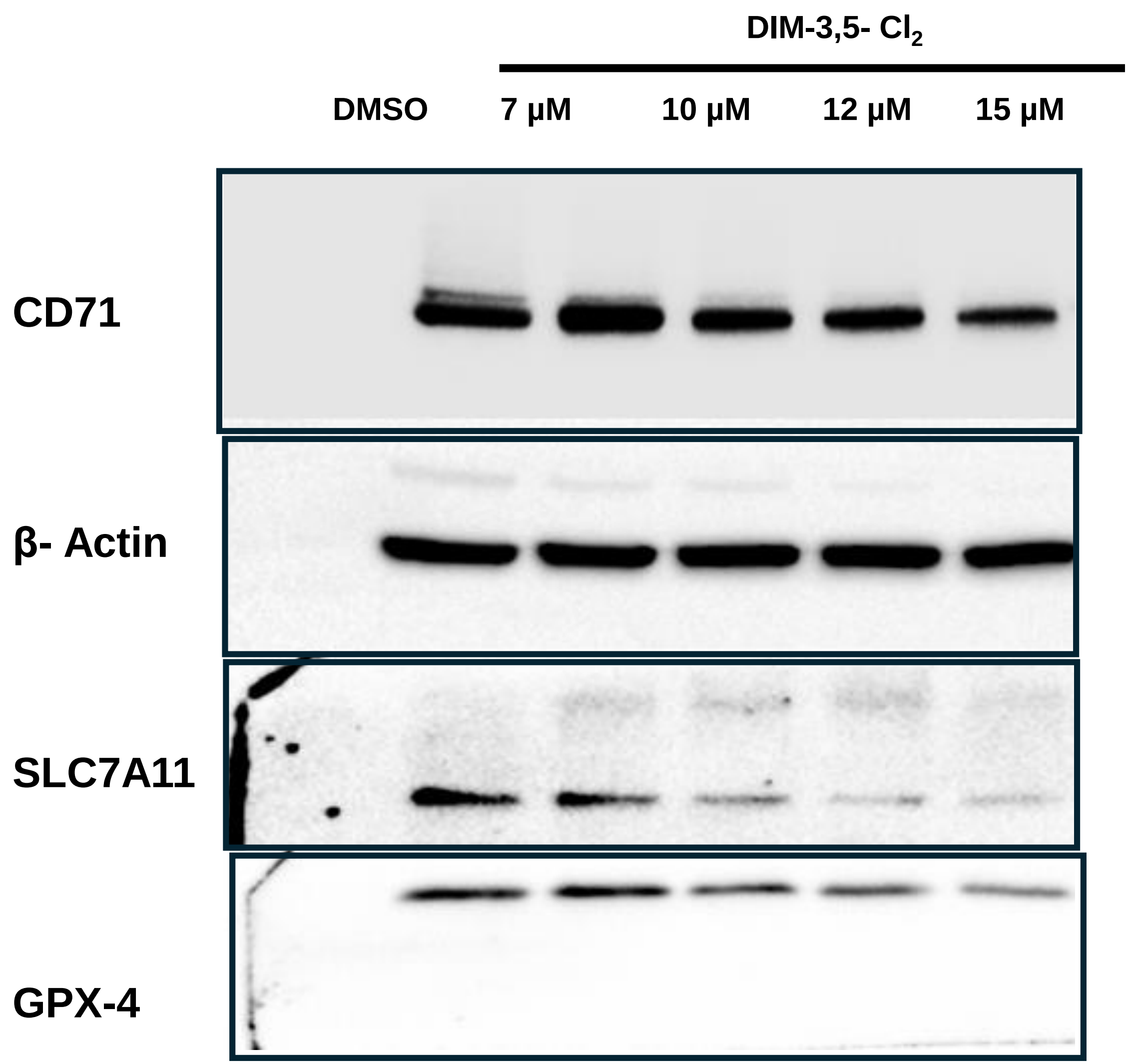

Figure 3B

24 hours
MDA-MB 468 cell

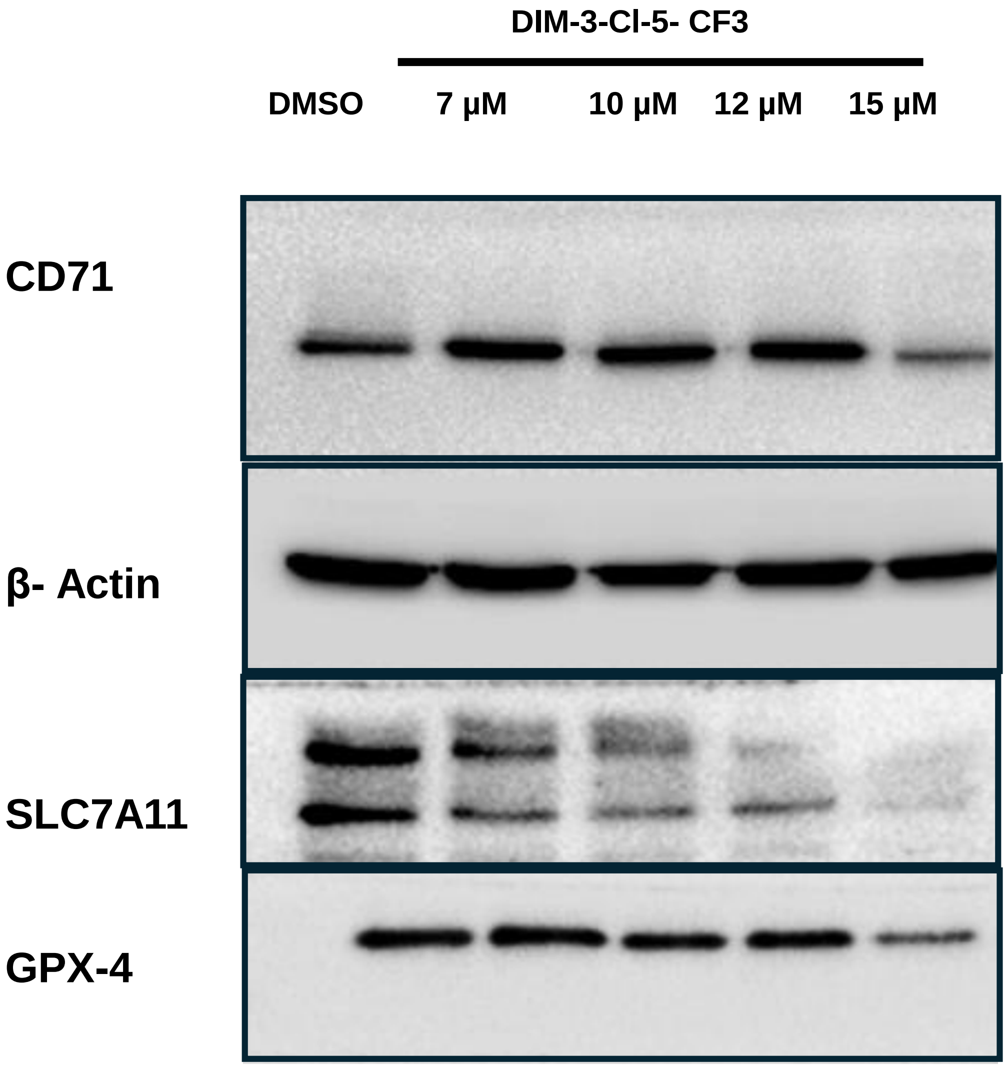

Figure 3C

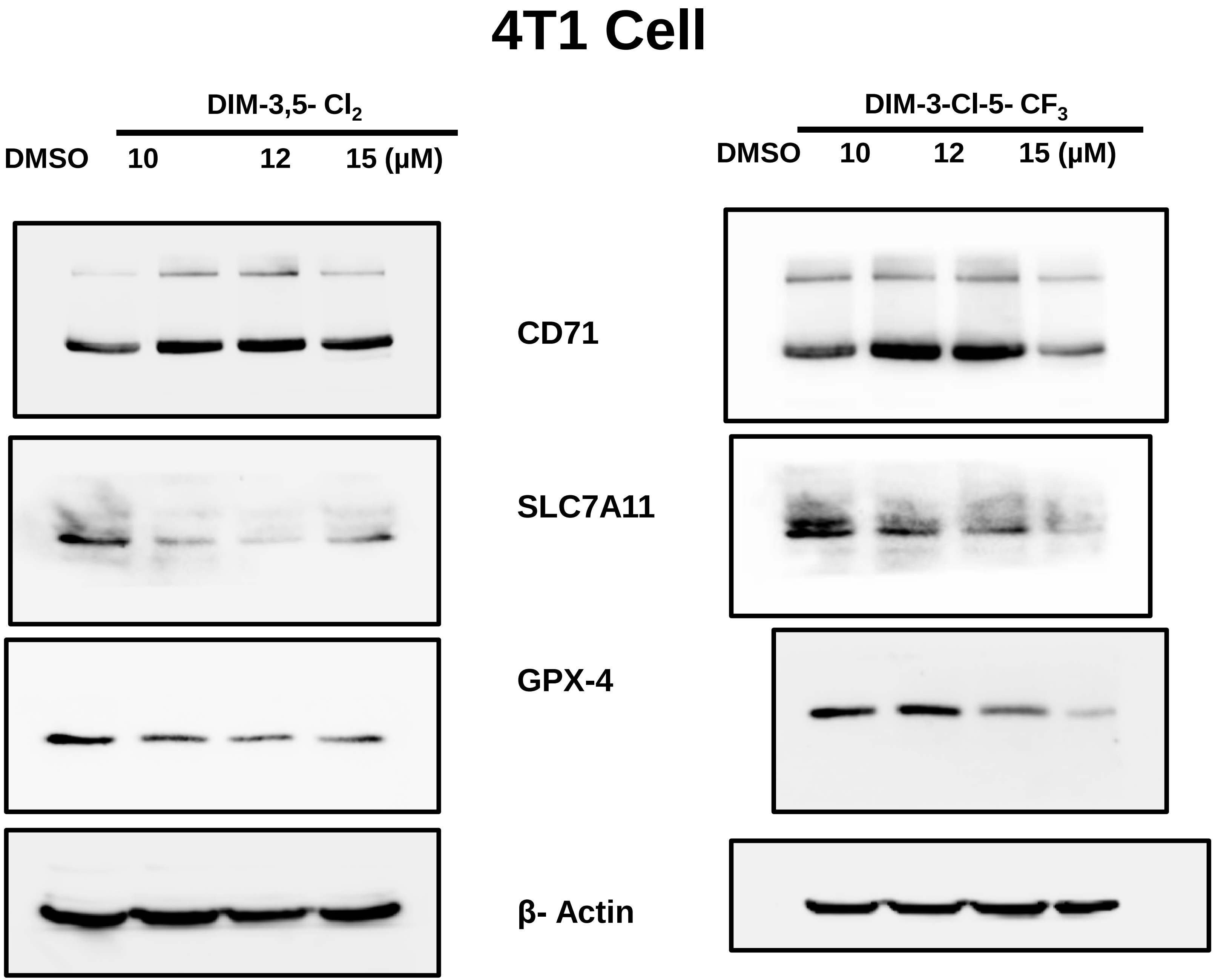
4T1 Cell
DIM-3,5- Cl2
DMSO 10 12 15 (µM)
DIM-3-Cl-5- CF3
DMSO 10 12 15 (µM)
CD71
SLC7A11
GPX-4
β- Actin

Figure 3C

4T1 Cell

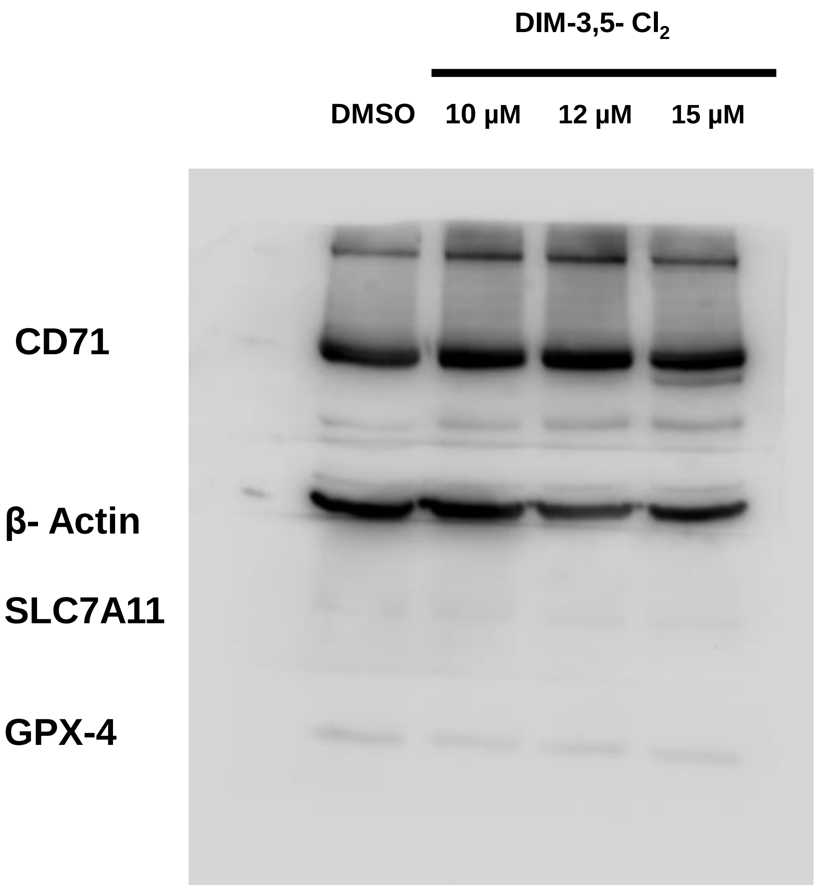

Full blot

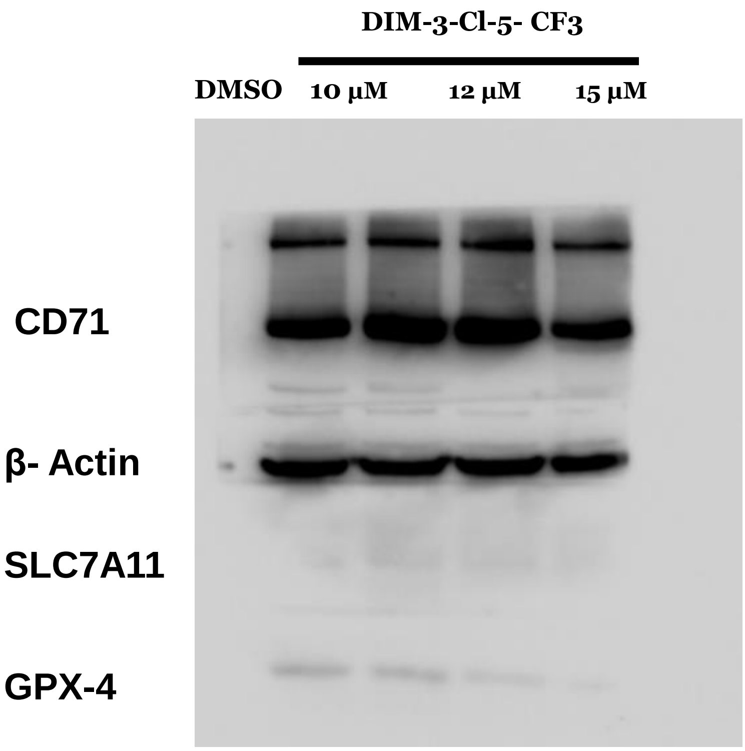

Full blot

Figure 3D

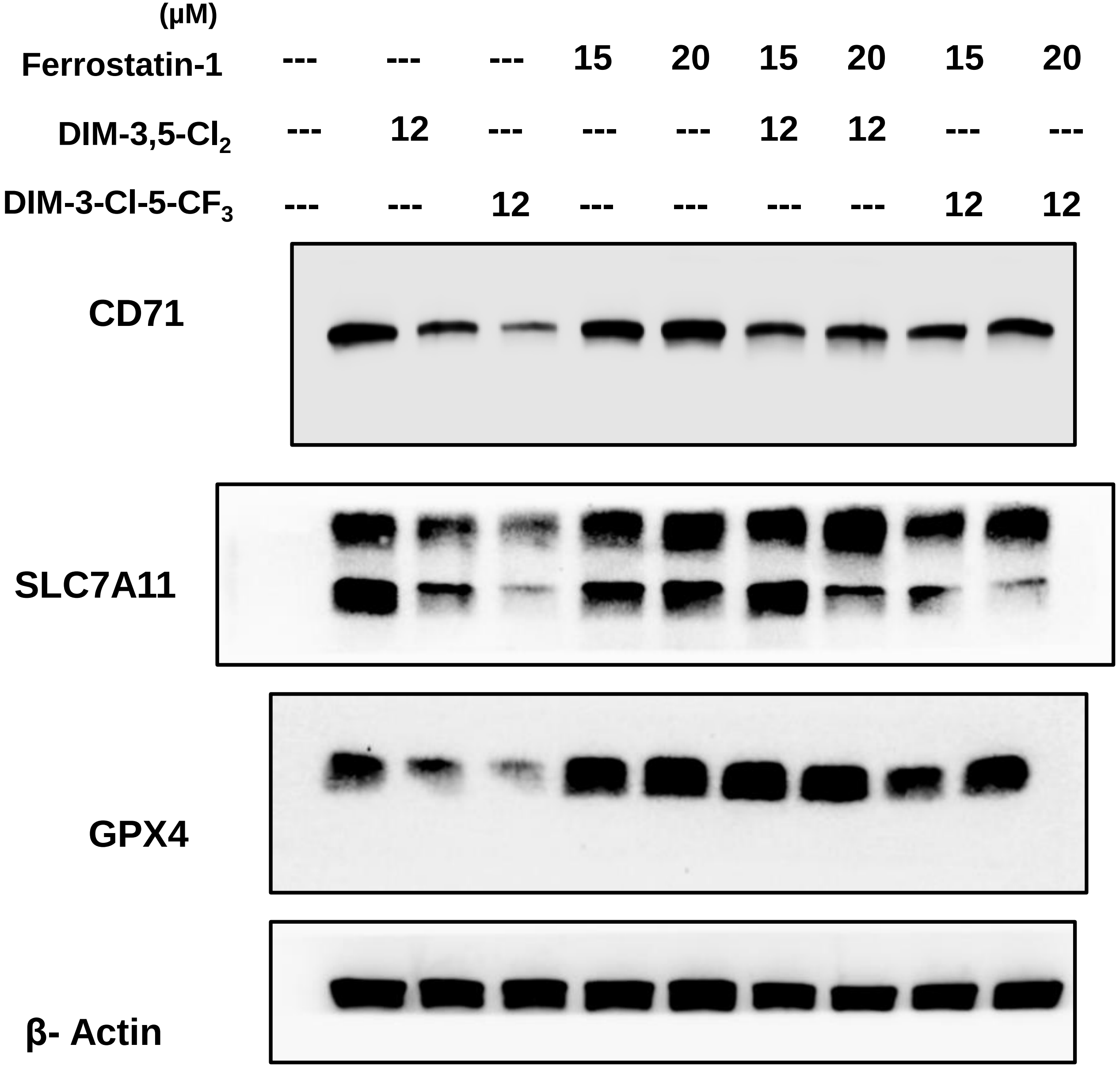

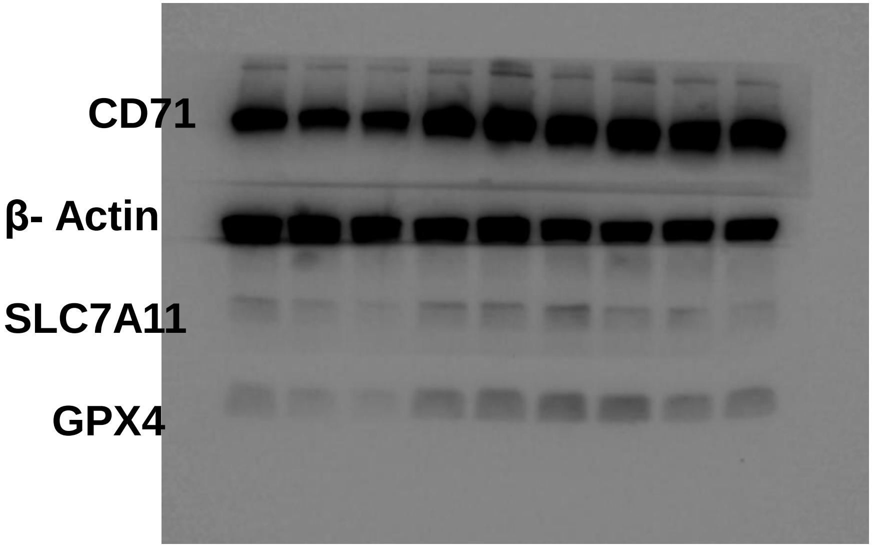

Full blot

Figure 3E

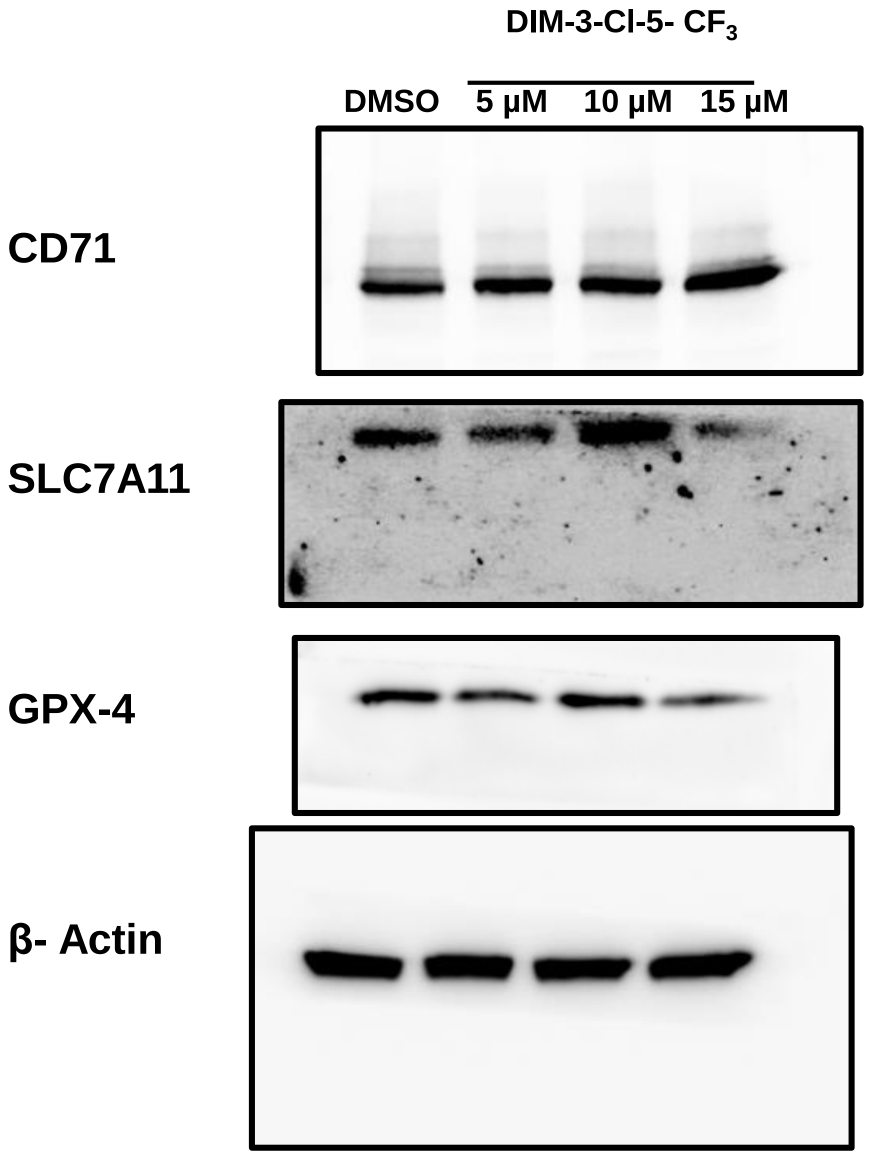

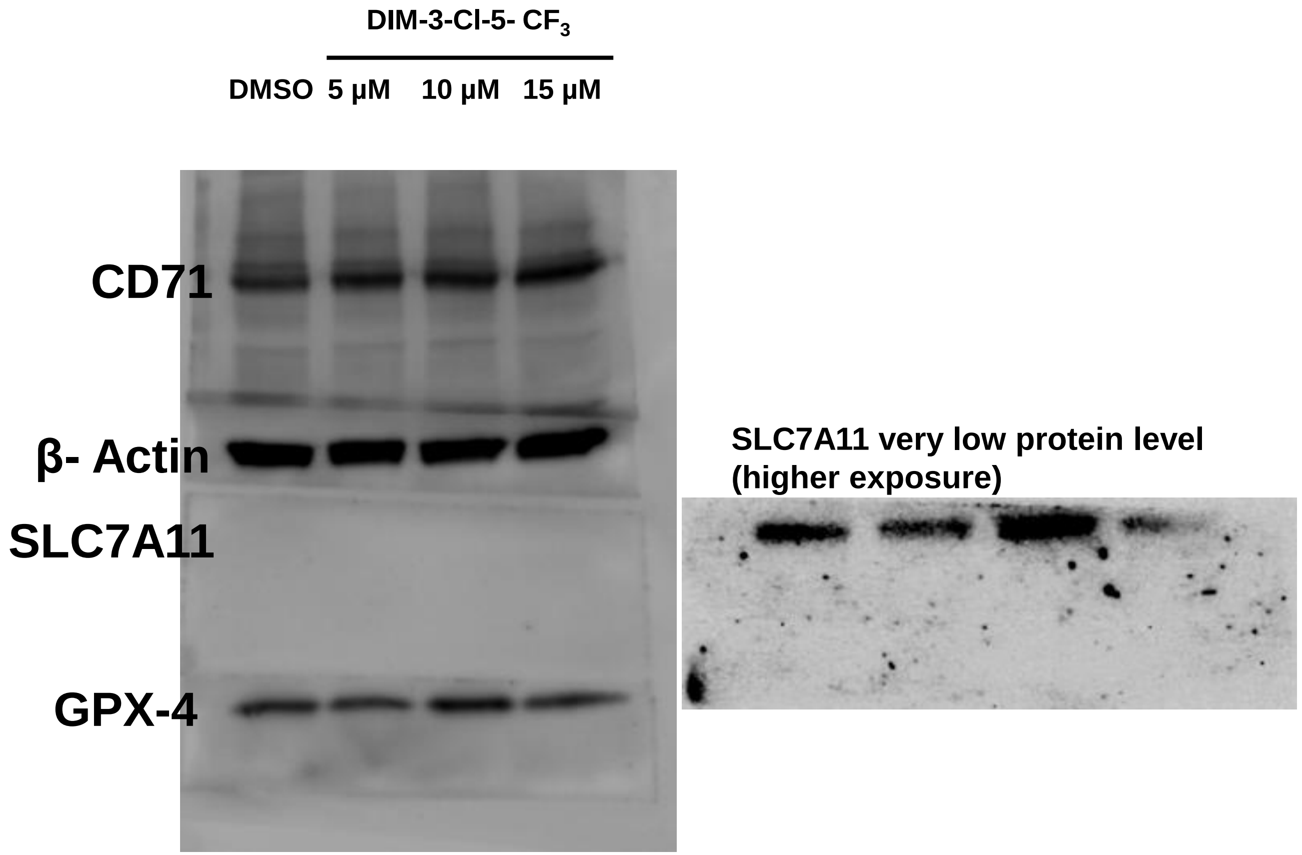

Full blot

Figure 3E

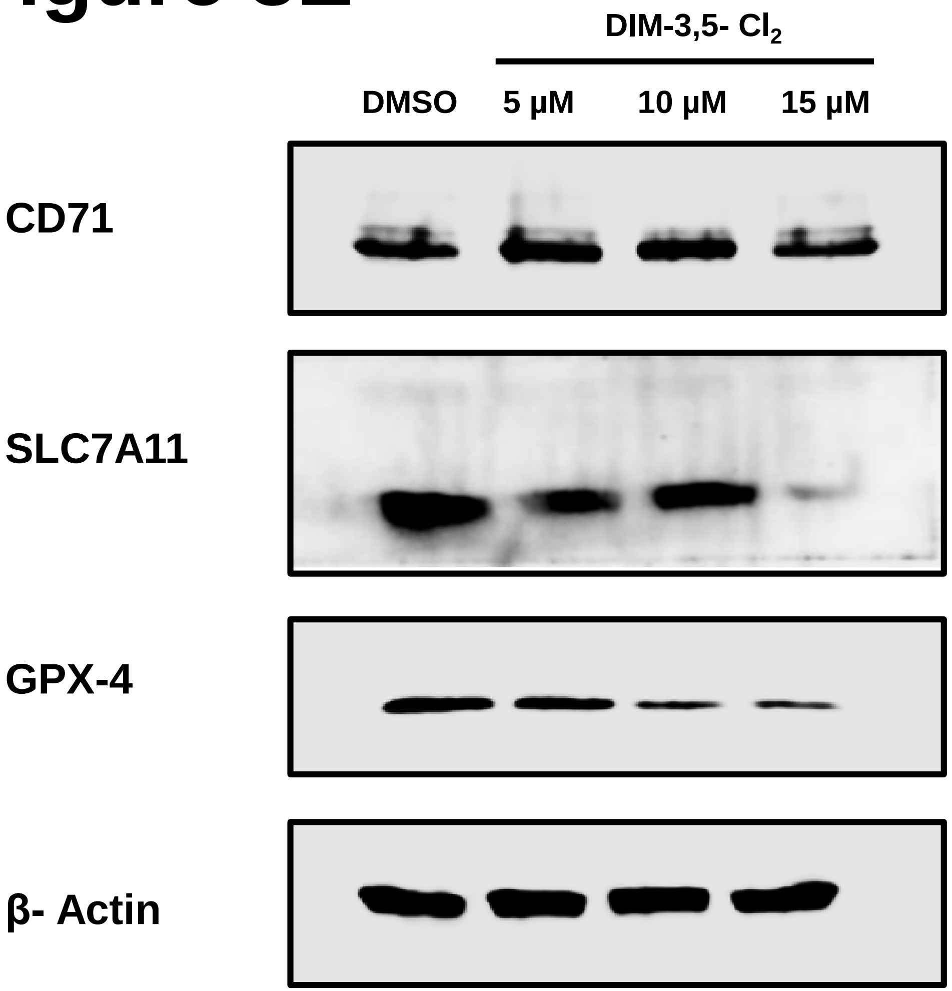

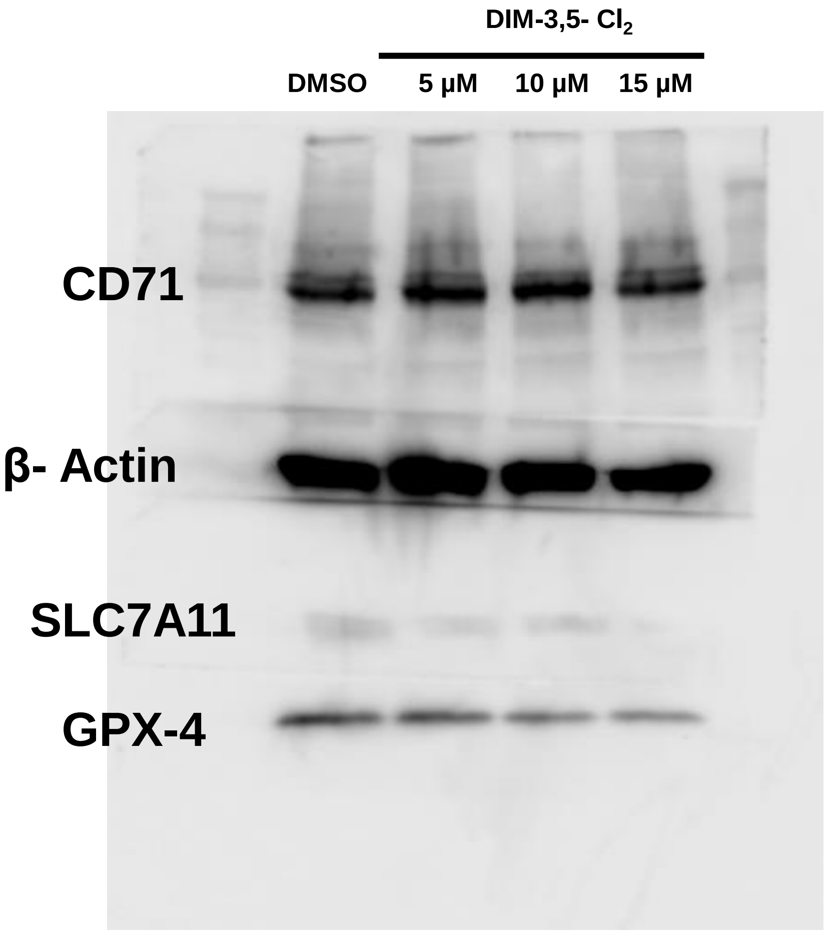

Full blot

Figure 4A

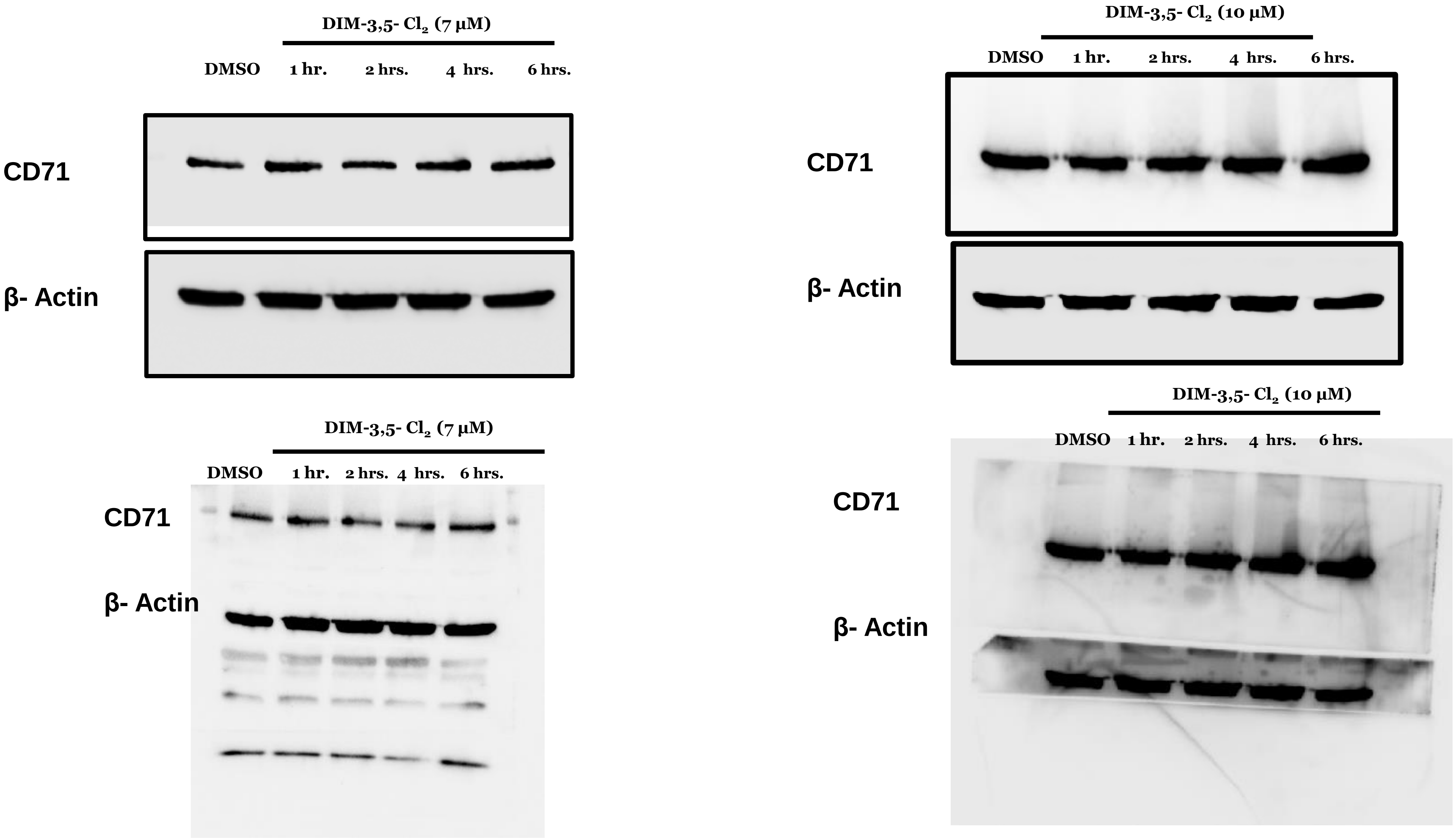

Full blot

Full blot

Figure 4B

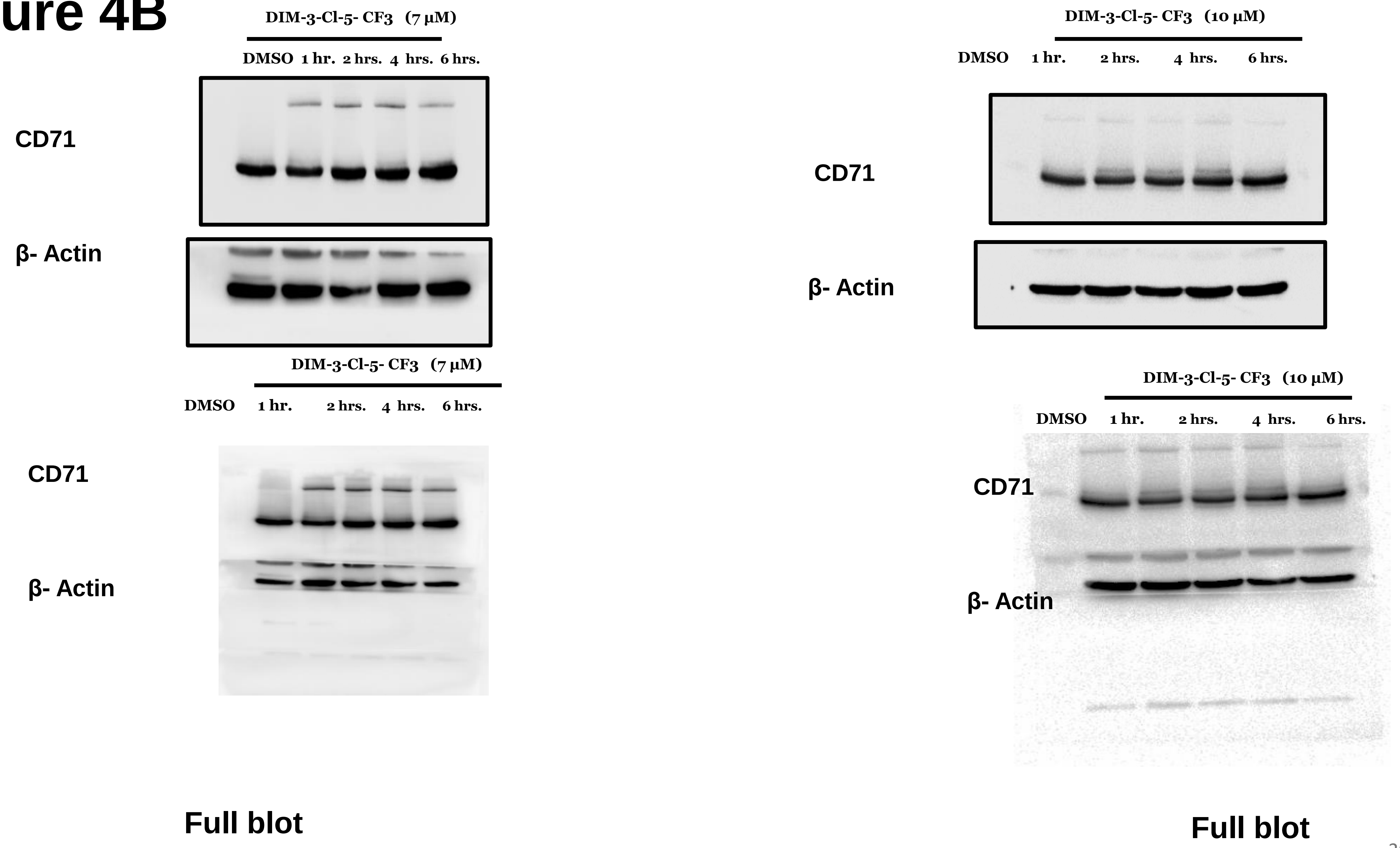
DIM-3-Cl-5- CF3 (7 µM)
DMSO 1 hr. 2 hrs. 4 hrs. 6 hrs.
CD71
β- Actin
DIM-3-Cl-5- CF3 (10 µM)
DMSO 1 hr. 2 hrs. 4 hrs. 6 hrs.
CD71
β- Actin
DIM-3-Cl-5- CF3 (7 µM)
DMSO 1 hr. 2 hrs. 4 hrs. 6 hrs.
CD71
β- Actin
Full blot
DIM-3-Cl-5- CF3 (10 µM)
DMSO 1 hr. 2 hrs. 4 hrs. 6 hrs.
CD71
β- Actin
Full blot

Figure 4E

PDxO model (BCM-HCI-3561)

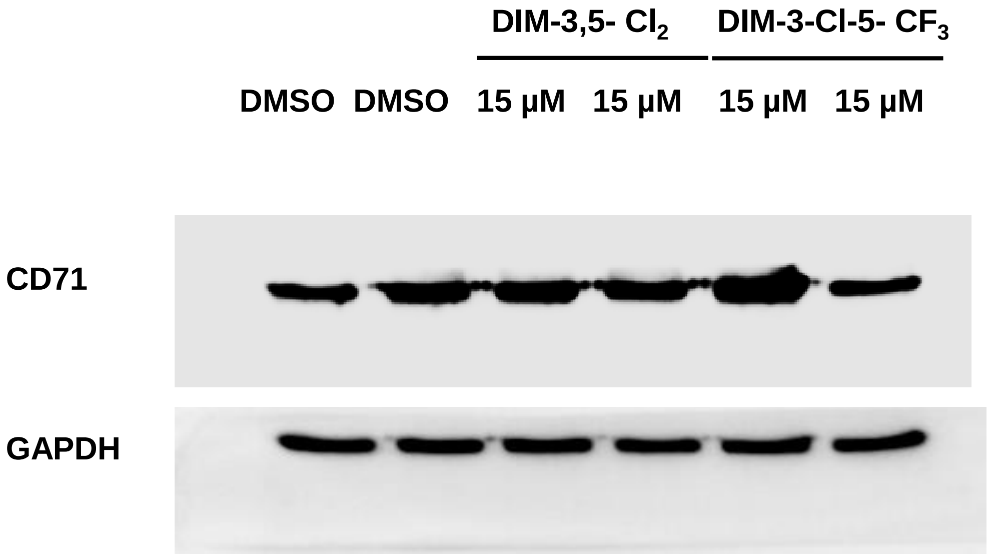

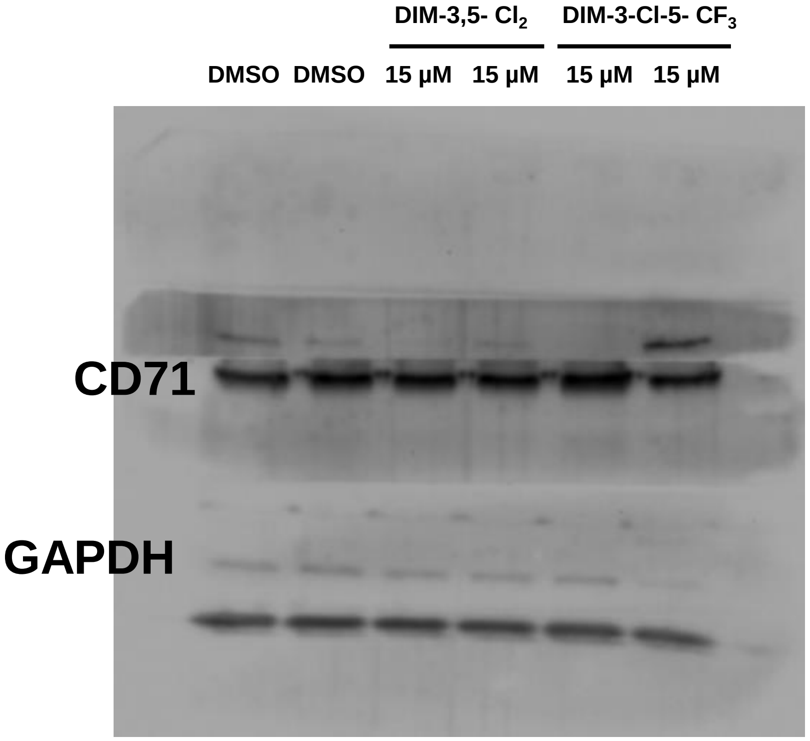

Full blot

Figure 4E

PDxO model (BCM-4175)

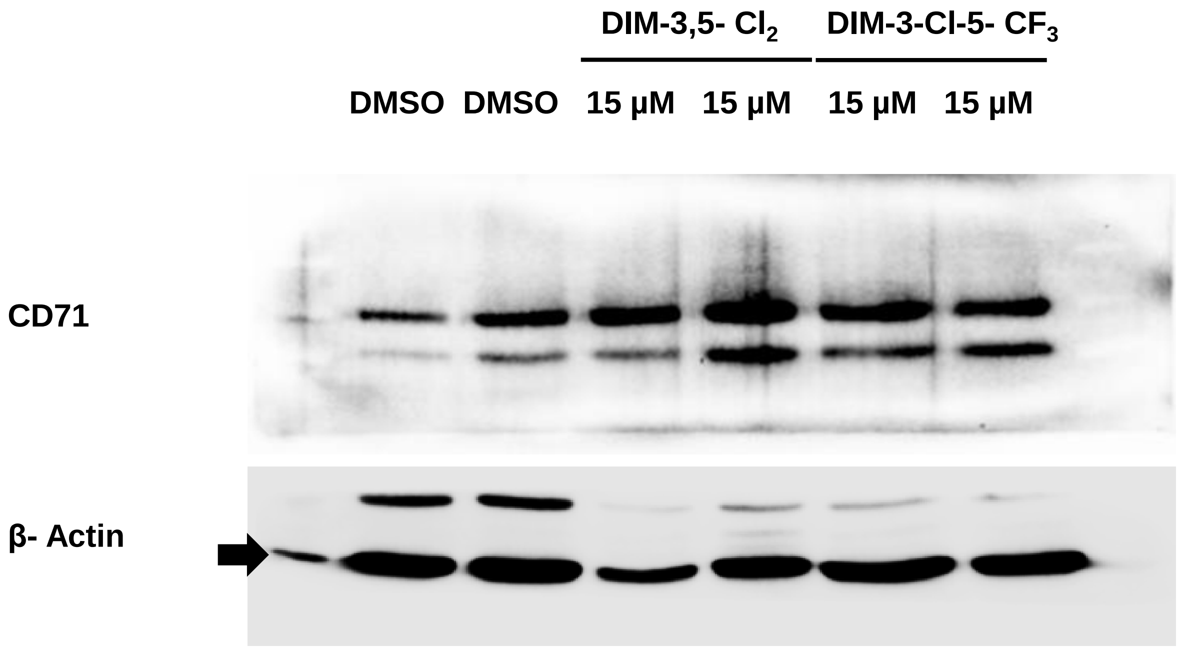

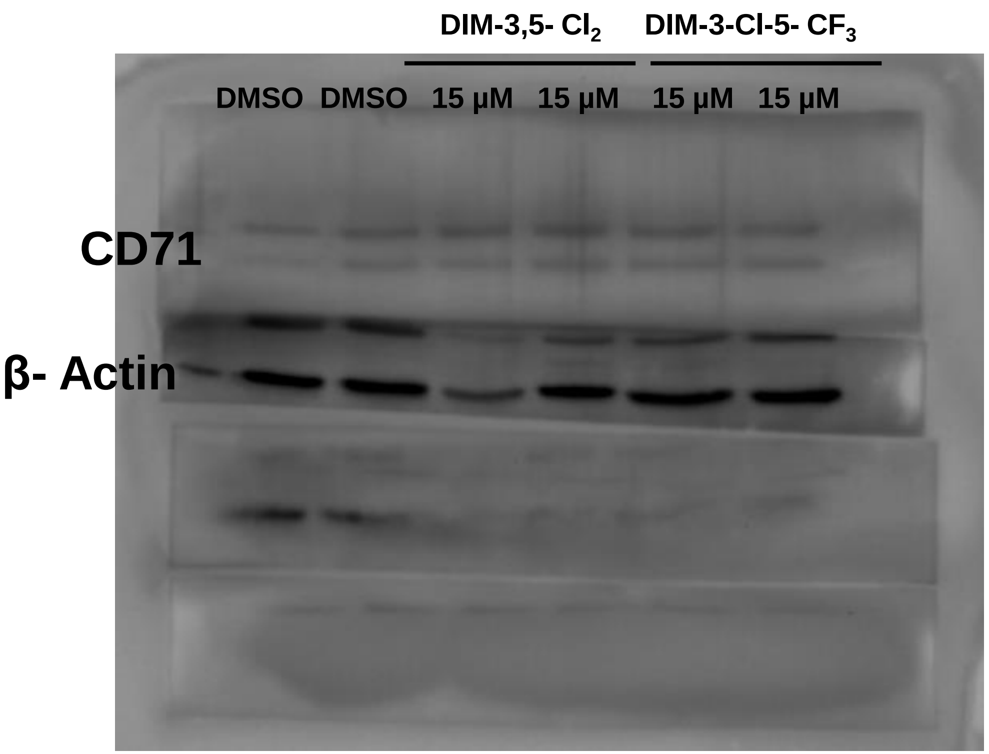

Full blot

Figure 4F

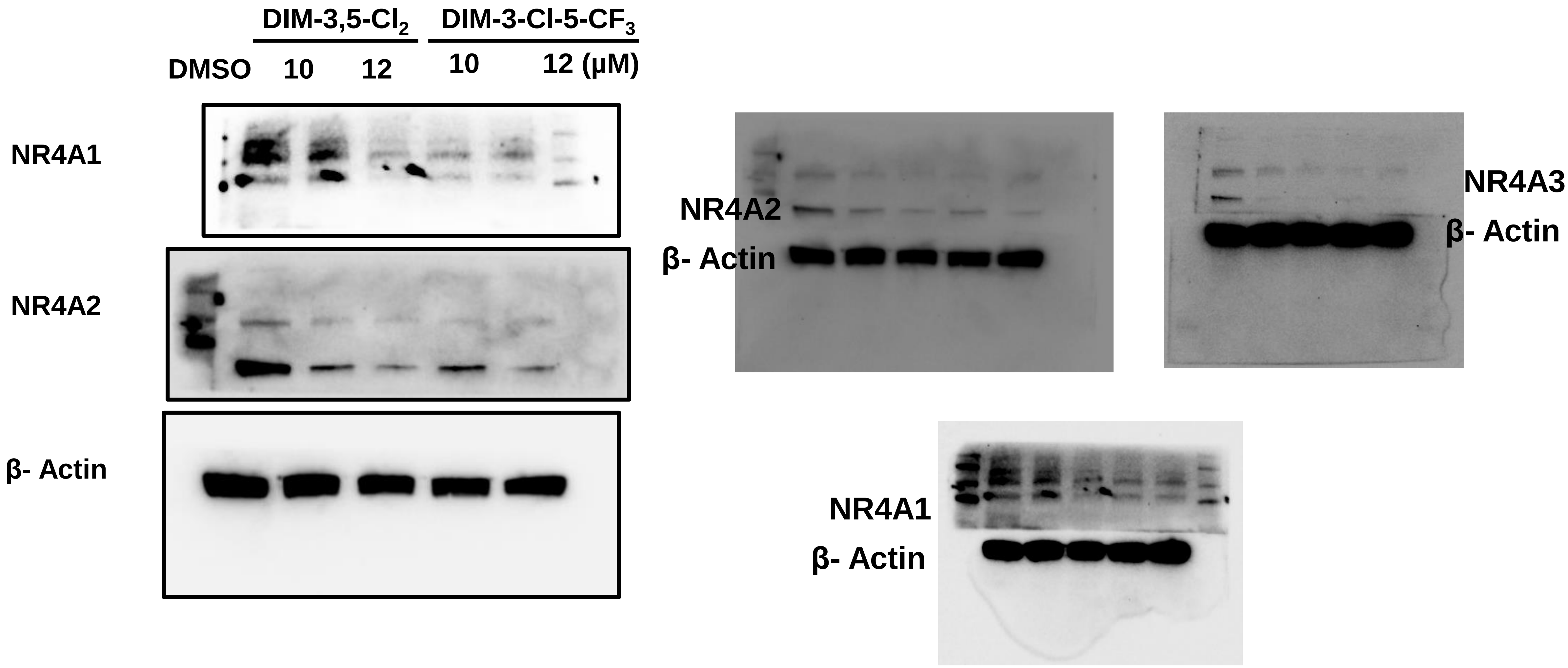

Full blot

Figure 5A

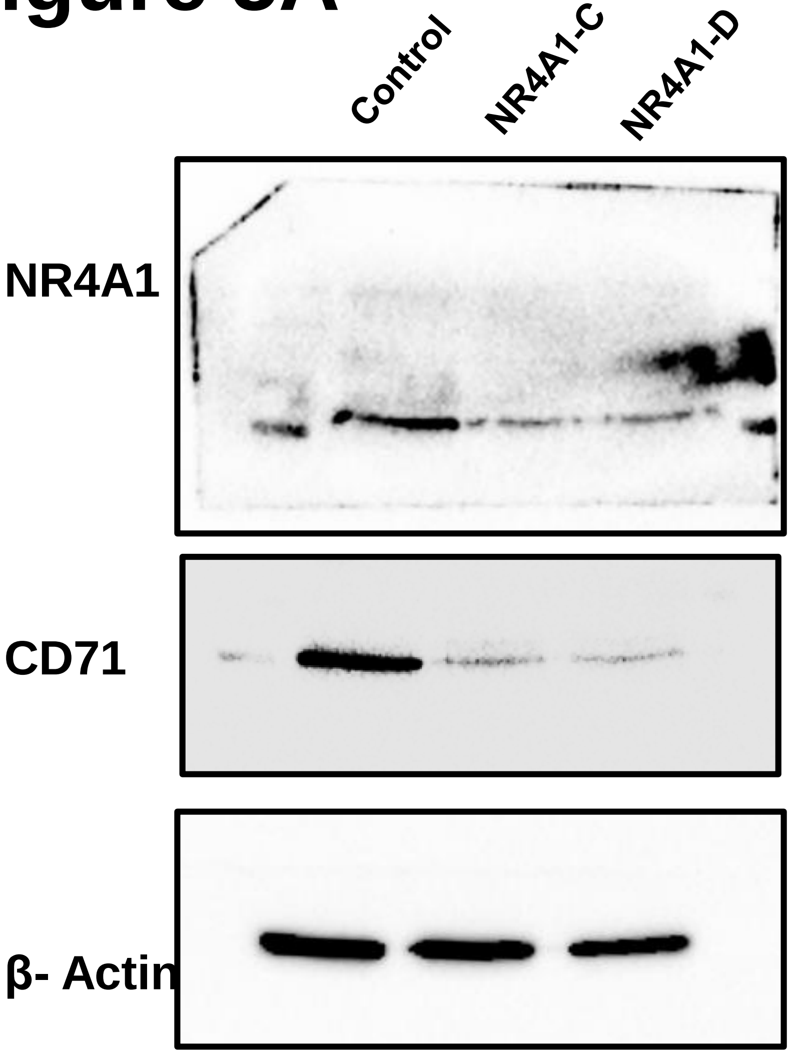

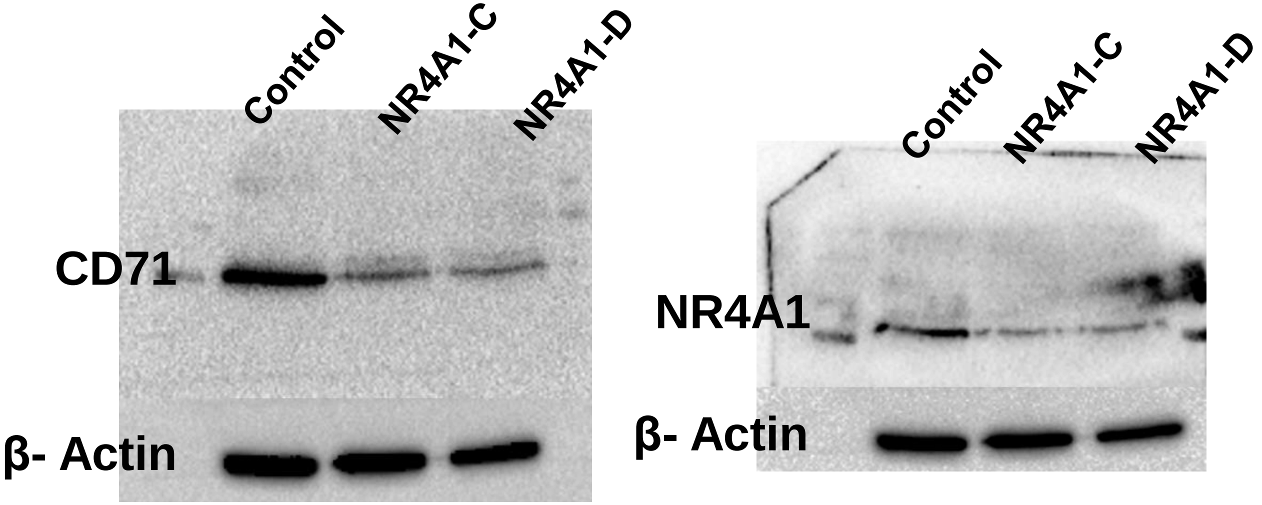

Full blot

Figure 5B

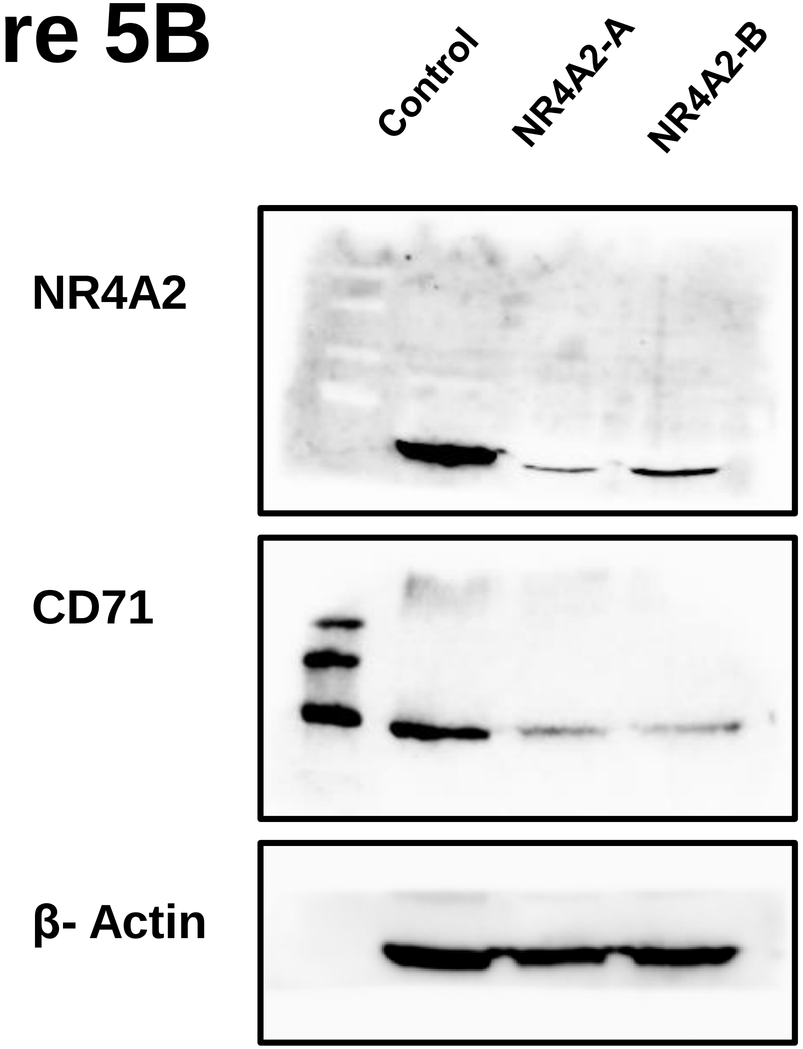

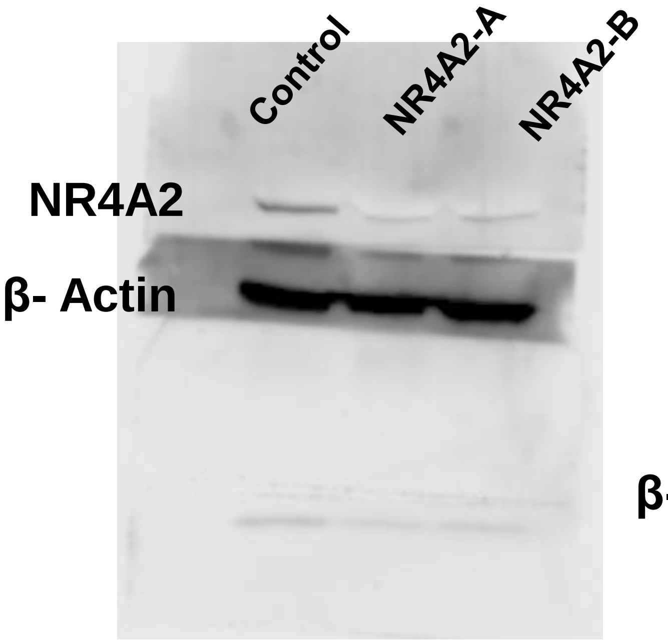

Full blot

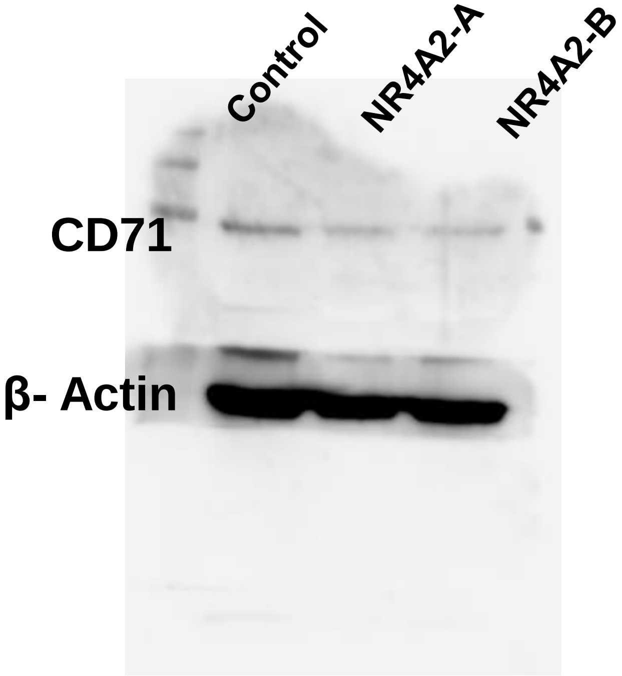

Full blot

Figure 5C

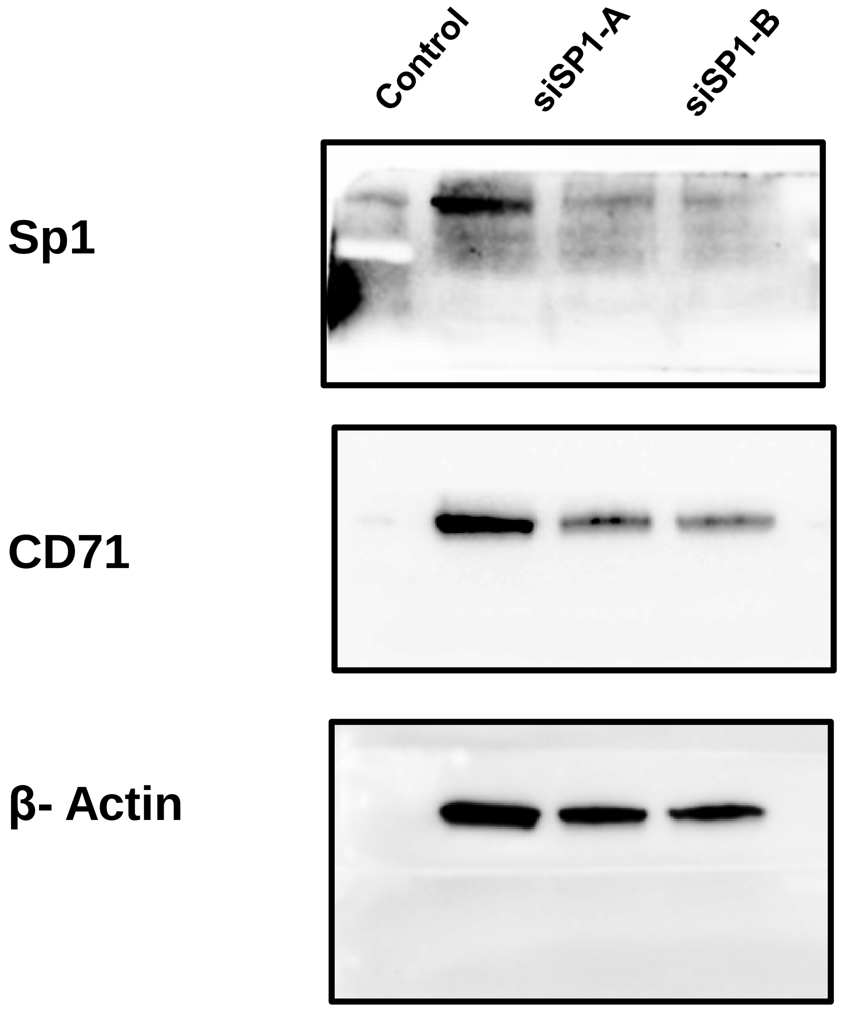

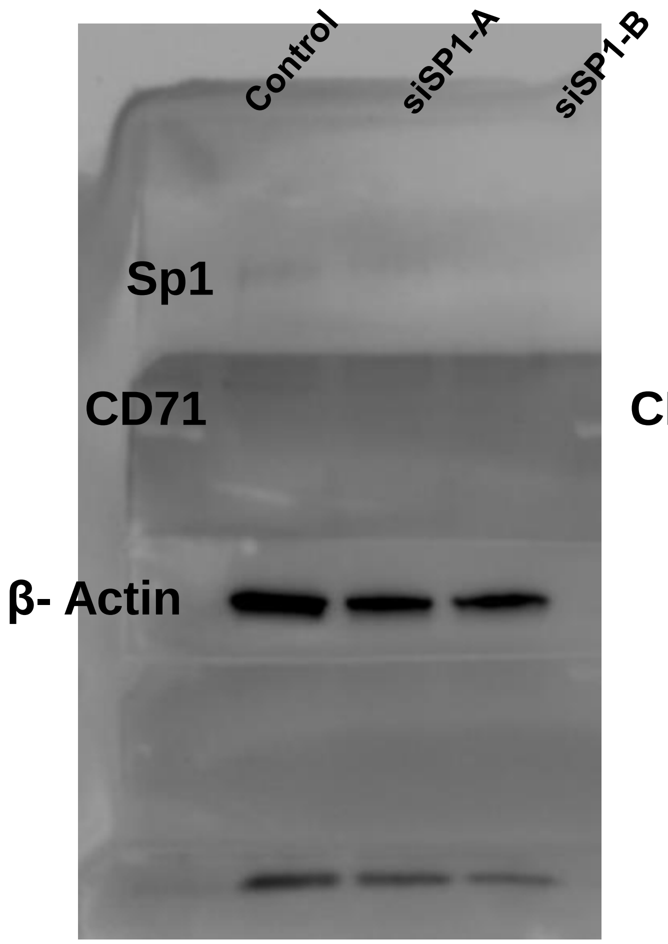

Full blot

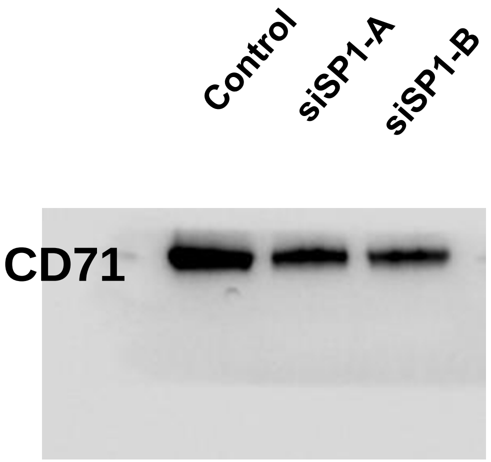

Full blot

Figure 5D

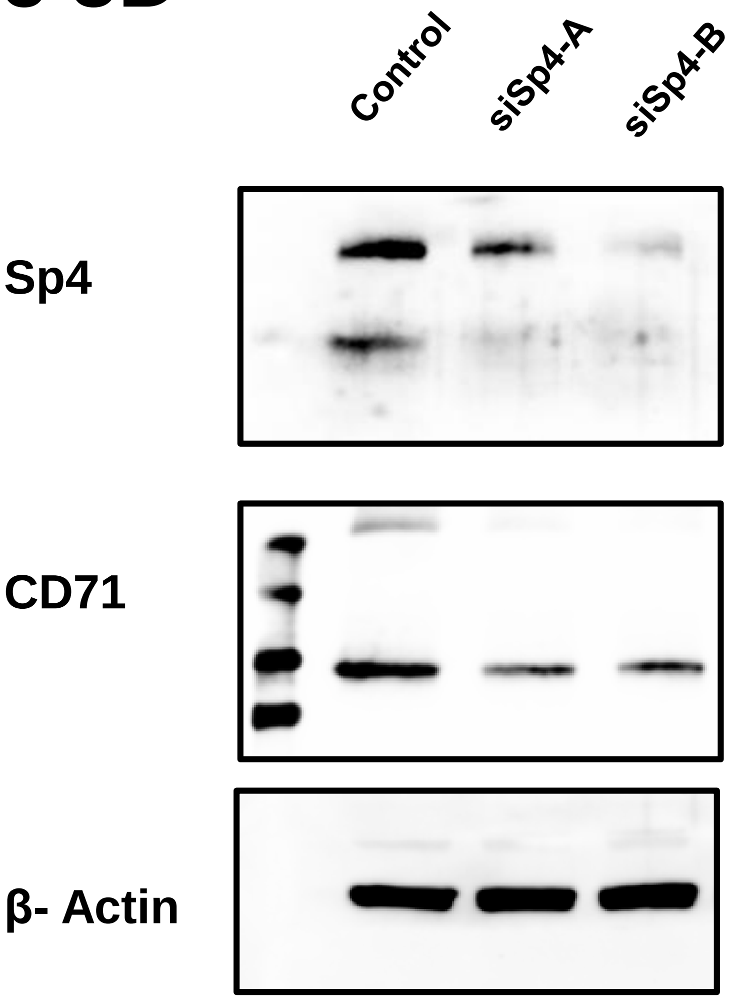

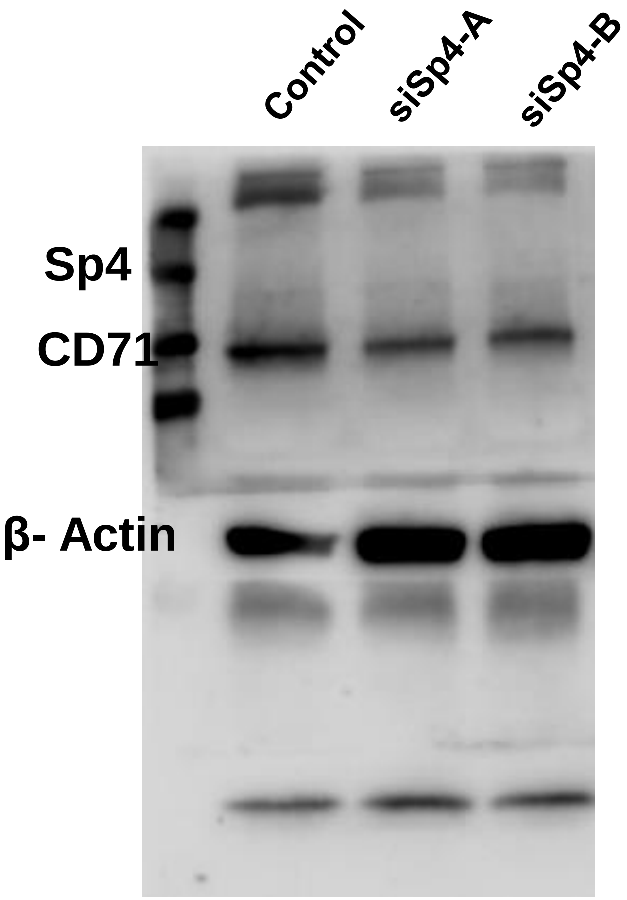

Full blot

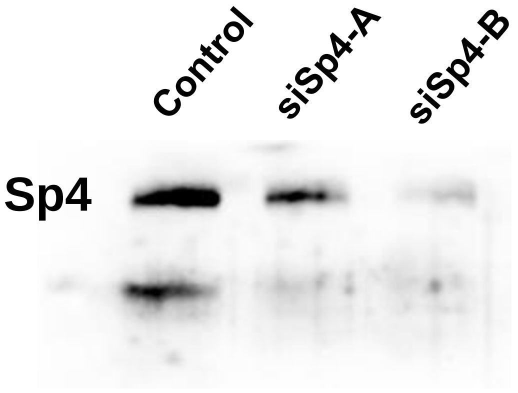

Full blot

Figure 5E

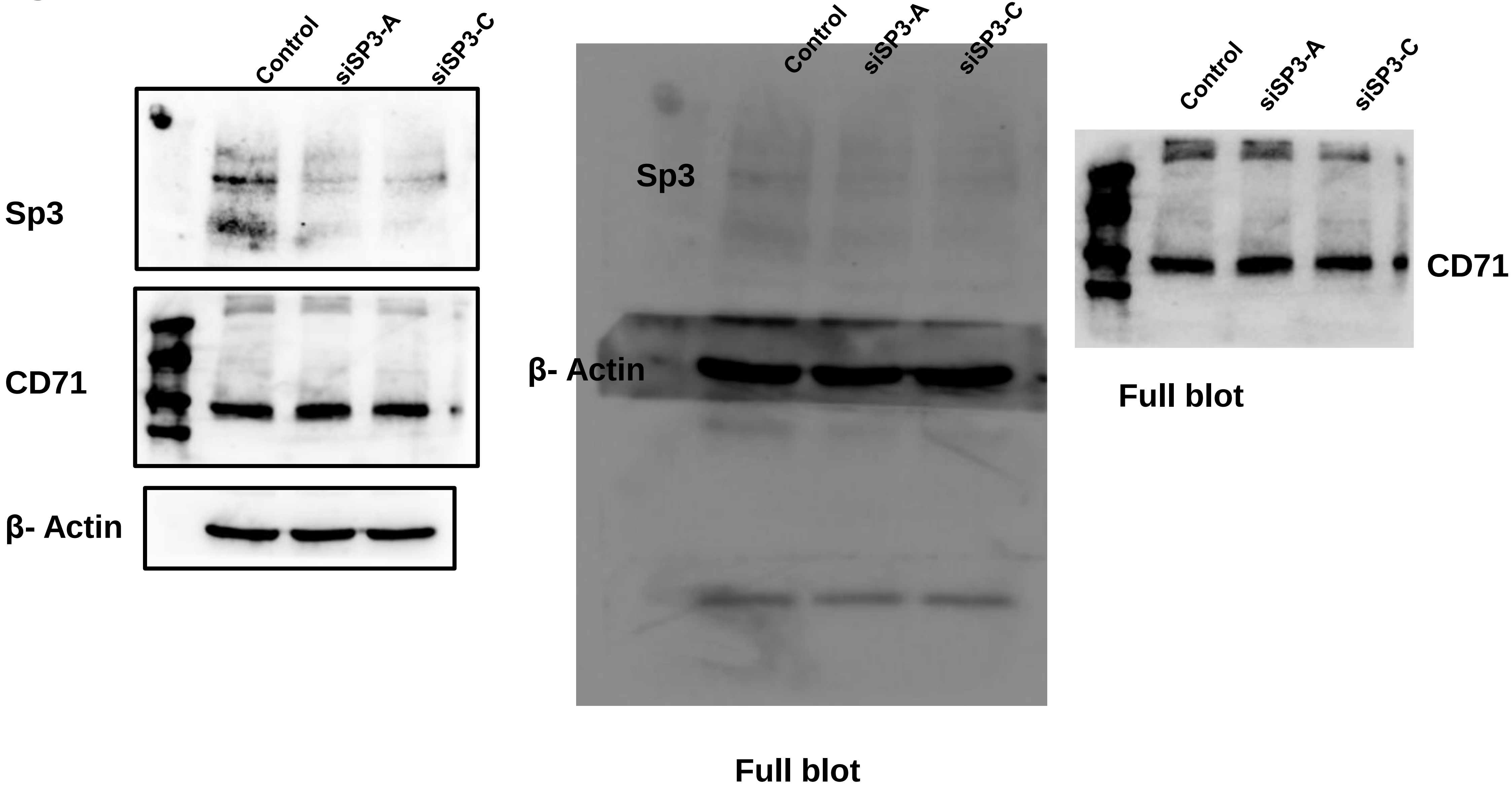
Control
siSP3-A
siSP3-C
Sp3
CD71
β- Actin
Control
siSP3-A
siSP3-C
Sp3
β- Actin
Full blot
Control
siSP3-A
siSP3-C
CD71
Full blot

Figure 5F

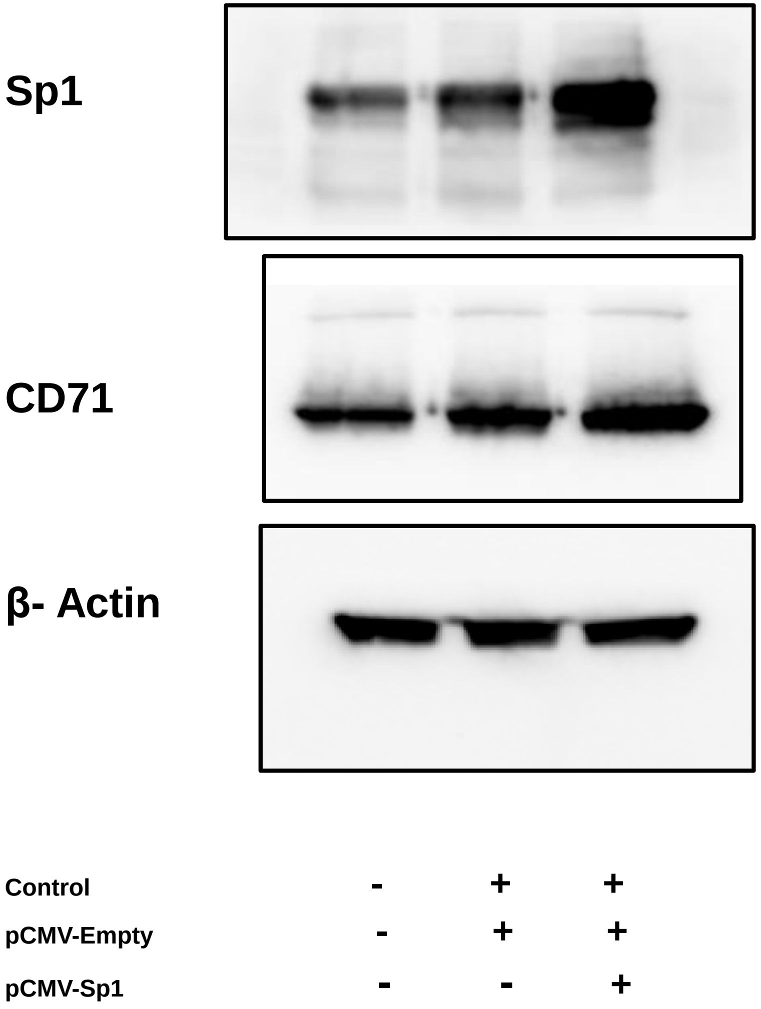

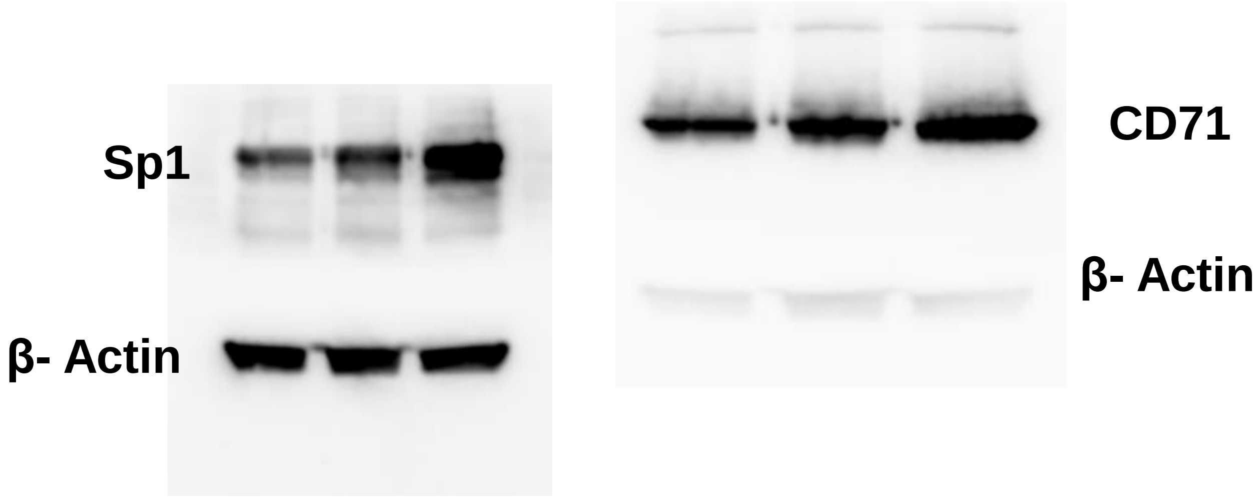

Full blot

Figure 5G

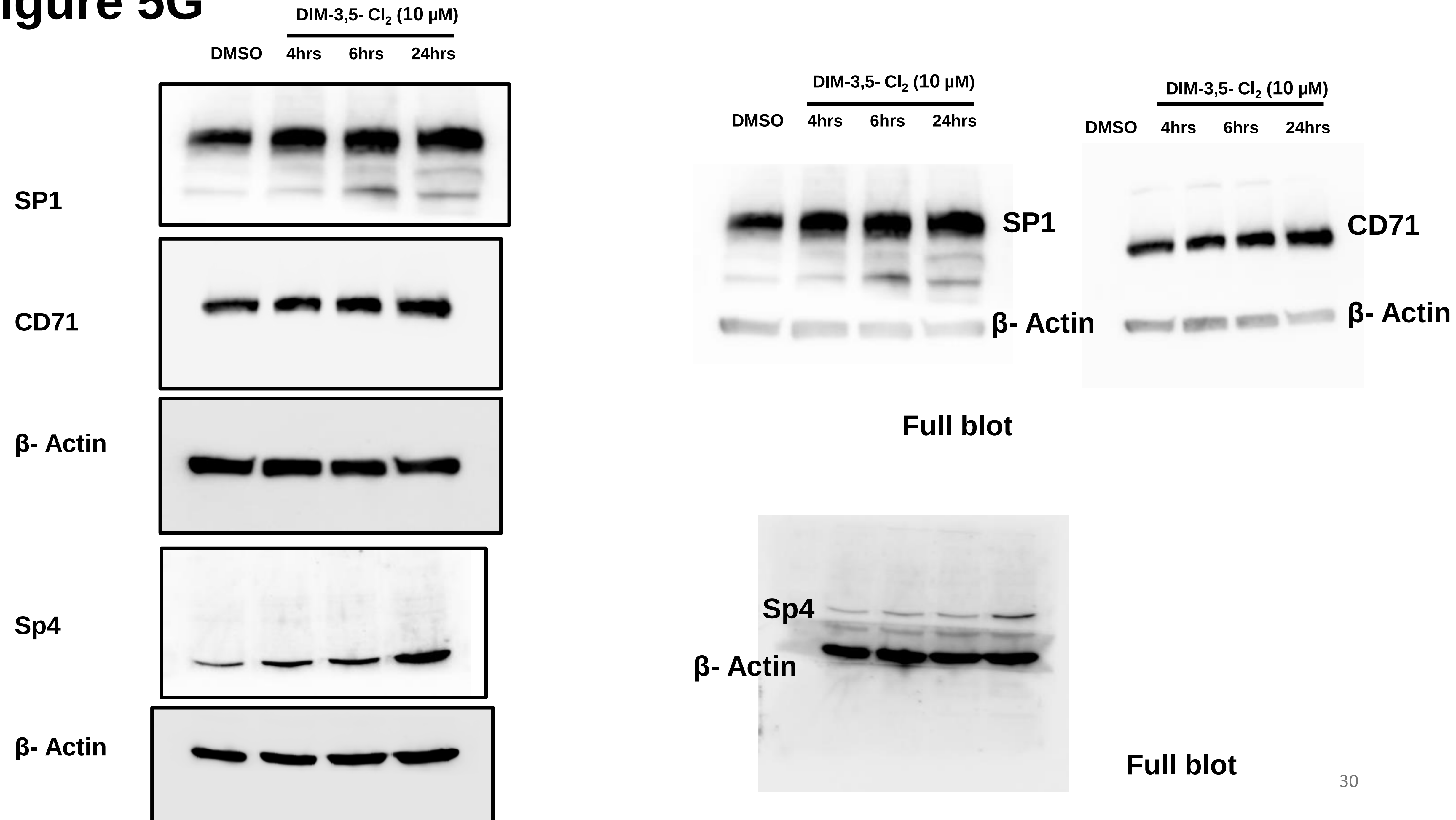
DIM-3,5- Cl2 (10 μM)
DMSO
4hrs
6hrs
24hrs
SP1
CD71
β- Actin
Sp4
β- Actin
DIM-3,5- Cl2 (10 μM)
DMSO
4hrs
6hrs
24hrs
SP1
β- Actin
DIM-3,5- Cl2 (10 μM)
DMSO
4hrs
6hrs
24hrs
CD71
β- Actin
Full blot
Sp4
β- Actin
Full blot

Figure 5H

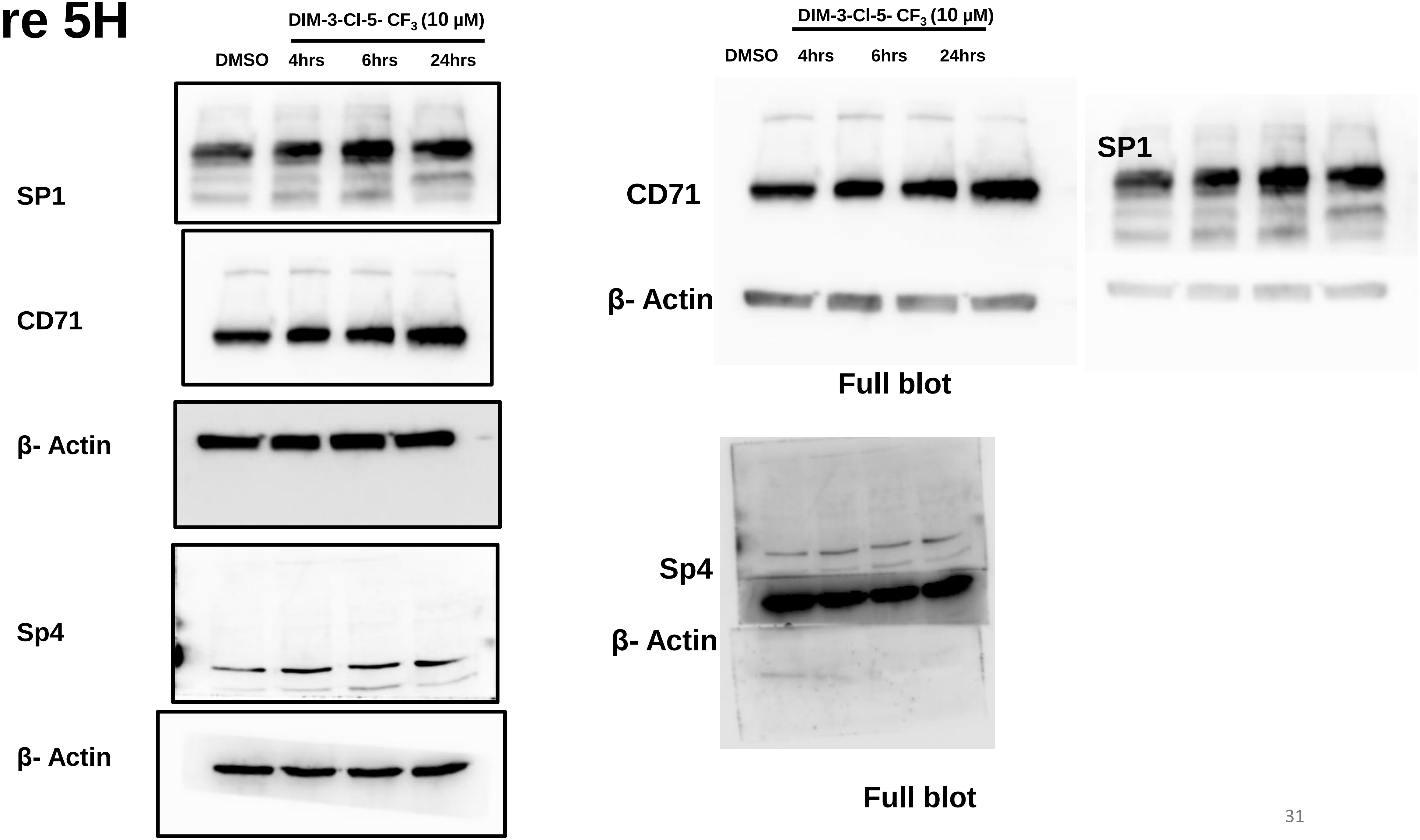
DIM-3-Cl-5- CF_3 (10 µM)
DMSO
4hrs
6hrs
24hrs
SP1
CD71
β- Actin
Sp4
β- Actin
DIM-3-Cl-5- CF_3 (10 µM)
DMSO
4hrs
6hrs
24hrs
CD71
β- Actin
SP1
Full blot
Sp4
β- Actin
Full blot

Supplementary Figure 01

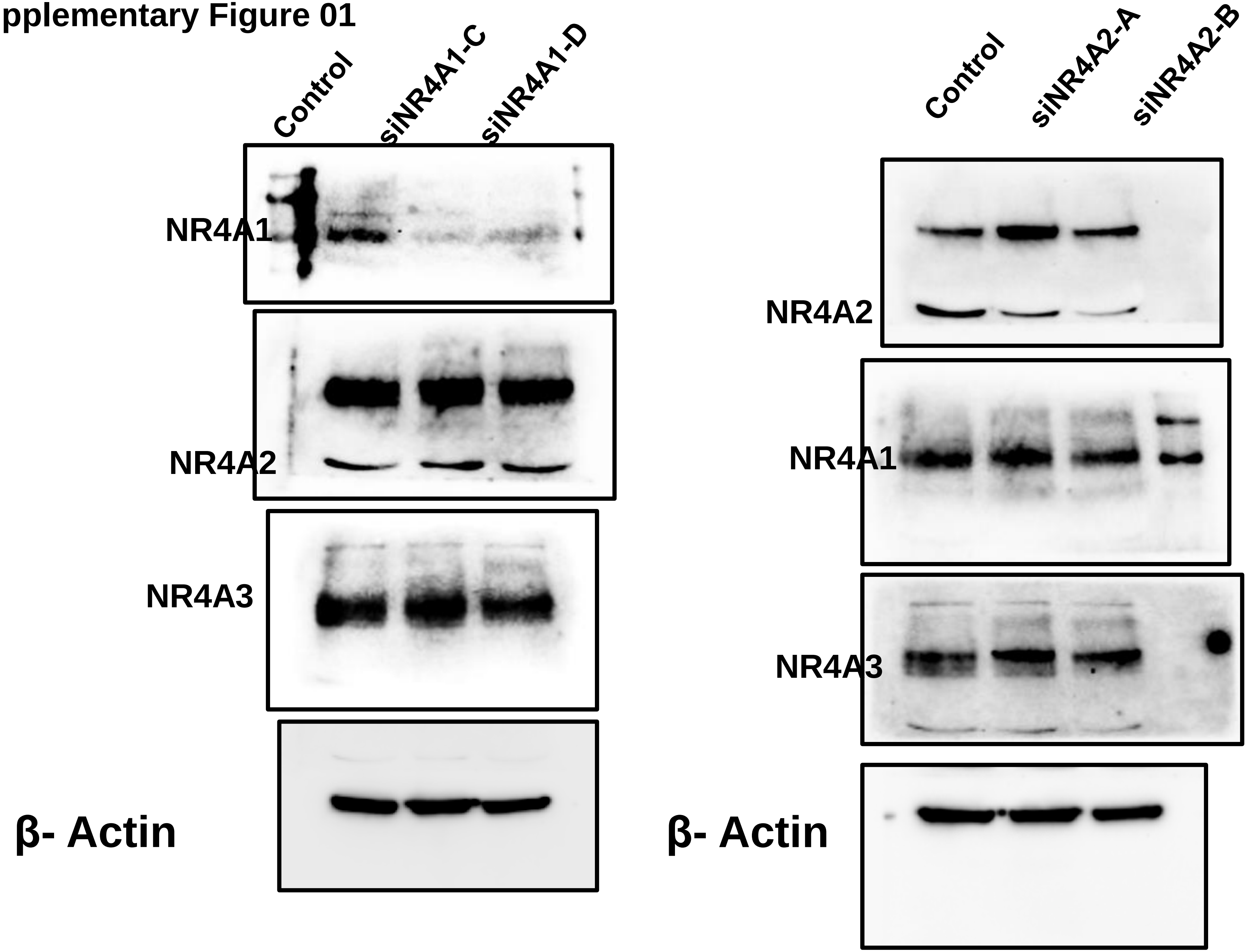

Supplement: Supplementary file 2 — Original Western Blots [file 41419_2025_8143_MOESM2_ESM.pdf]
